# Application of visible-light photosensitization to form alkyl-radical-derived thin films on gold

**DOI:** 10.3762/bjnano.8.187

**Published:** 2017-09-06

**Authors:** Rashanique D Quarels, Xianglin Zhai, Neepa Kuruppu, Jenny K Hedlund, Ashley A Ellsworth, Amy V Walker, Jayne C Garno, Justin R Ragains

**Affiliations:** 1 Department of Chemistry, Louisiana State University, 232 Choppin Hall, Baton Rouge, LA, 70803, USA; 2 Department of Chemistry and Biochemistry, University of Texas at Dallas, 800 W. Campbell Rd., Richardson, TX, 75080, USA; 3 Department of Materials Science, University of Texas at Dallas, 800 W. Campbell Rd., Richardson, TX, 75080, USA

**Keywords:** atomic force microscopy, organic thin film, particle lithography, photosensitization, TOF SIMS

## Abstract

Visible-light irradiation of phthalimide esters in the presence of the photosensitizer [Ru(bpy)_3_]^2+^ and the stoichiometric reducing agent benzyl nicotinamide results in the formation of alkyl radicals under mild conditions. This approach to radical generation has proven useful for the synthesis of small organic molecules. Herein, we demonstrate for the first time the visible-light photosensitized deposition of robust alkyl thin films on Au surfaces using phthalimide esters as the alkyl radical precursors. In particular, we combine visible-light photosensitization with particle lithography to produce nanostructured thin films, the thickness of which can be measured easily using AFM cursor profiles. Analysis with AFM demonstrated that the films are robust and resistant to mechanical force while contact angle goniometry suggests a multilayered and disordered film structure. Analysis with IRRAS, XPS, and TOF SIMS provides further insights.

## Introduction

The deposition of radical-derived organic thin films has emerged as an attractive alternative to the grafting of molecules such as thiols, chlorosilanes and alkoxysilanes [1–28]. Carbon-centered radicals have proven to be versatile grafting species that can covalently bond to a number of surfaces including precious, coinage and industrial metals [1–6], hydrogen-terminated silicon [7–8], and indium tin oxide [9–10]. The resulting aryl- and alkyl-radical-derived thin films are resistant toward oxidation, chemical treatment, heating, and mechanical force.

The most popular method for the deposition of radical-derived films involves the electrografting of arenediazonium salts [1–2,6–14,17–20]. An arenediazonium ion can accept a single electron from a cathode to generate aryl radical and N_2_ at relatively high potentials. Rapid covalent bonding [11–14] of aryl radical to surfaces followed by further attachment of radicals to already-grafted arenes results in polymerization and generates dense polyphenylene multilayers. Other methods for aryl radical grafting include electrografting of diaryliodonium salts [15–16], and dimethoxybenzene- or [Ru(bpy)_3_]^2+^-photosensitized conversion of arenediazonium salts to aryl radicals in the presence of metal and polymer surfaces [17–20]. As such, arenediazonium salts are workhorses for the growth of aryl radical-derived thin films with broad applications.

In stark contrast, alkyl radical-derived thin film growth has received much less attention but is attractive given the broad utility of alkyl and alkyl-containing films [3–5,21–28]. This is due to limitations associated with alkyl radical precursors. Thin films have been deposited starting from alkyl radical precursors including carboxylic acids [2,22–23], Grignard reagents [2,24–27], and alkyl halides [2–5,28] using electrografting techniques. Electrografting techniques involving the deposition of radical-derived thin films from these precursors suffer from limitations including overoxidation of radical intermediates to carbocations (in the case of carboxylic acids) [2], moisture sensitivity (in the case of Grignard reagents), and spontaneous grafting of the precursor (in the case of alkyl halides) [29–32].

**Scheme 1:**
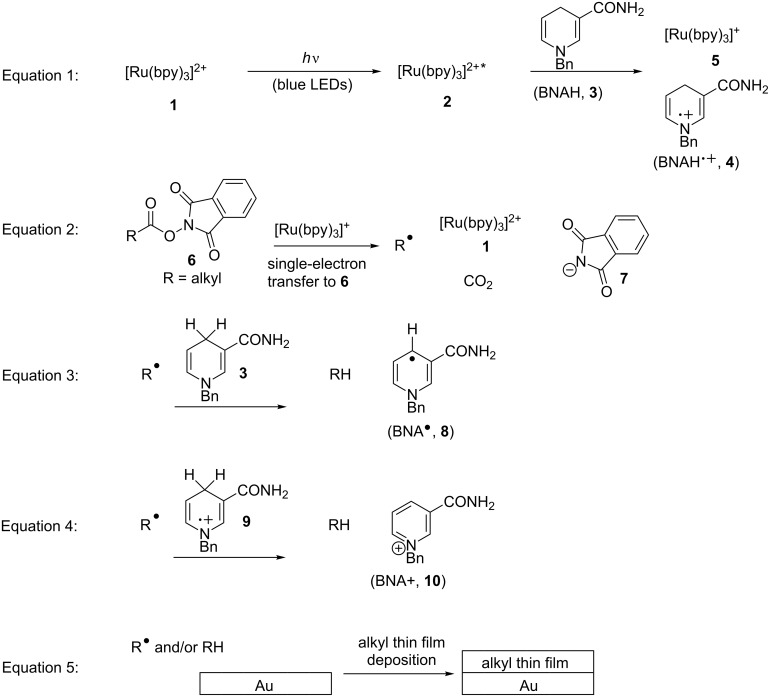
Proposed visible-light-promoted thin film deposition on Au surface.

We envisioned an alternative approach to alkyl radical-derived thin film growth that occurs under mildly reducing conditions and does not suffer from moisture sensitivity. This involves phthalimide esters (**6**, Equation 2, Scheme 1), species that are synthesized in one step from carboxylic acids [33–40] and which have been used to address a number of difficult problems in organic synthesis [36–40]. Therefore (Scheme 1), visible-light irradiation (e.g., with blue LEDs) of the photosensitizer [Ru(bpy)_3_]^2+^ (**1**) results in a long-lived (τ = 1100 ns) [41], oxidizing metal-to-ligand charge transfer excited state [Ru(bpy)_3_]^2+*^ (**2**, *E*_1/2(M*/M-)_ = +0.77 V, SCE) [41] that accepts an electron from benzyl nicotinamide (BNAH, **3**, *E*_1/2_ = +0.76 V, SCE) [41] to generate the strongly-reducing [Ru(bpy)_3_]^+^ (**5**) and benzyl nicotinamide radical cation (BNAH**^∙^**^+^, **4**, Equation 1). Irreversible single-electron transfer from [Ru(bpy)_3_]^+^ (**5**, *E*_1/2(M/M-)_ = −1.33 V, SCE) [41] to **6** (*E*_1/2_ ≈ −1.3 V, SCE) [37] leads to a short-lived radical anion [37] that fragments to CO_2_, phthalimide anion **7**, and alkyl radical (**R^∙^**) and turns over the photosensitizer (Equation 2) [39]. The fate of **7** is protonation, possibly by BNAH**^∙^**^+^ (**4**), to generate phthalimide [39]. Alkyl radical **R^∙^** would then be subject to processes including reduction to alkane RH with generation of benzyl nicotinamide radical (BNA**^∙^**, **8**, Equation 3) or fully oxidized pyridinium BNA^+^ (**10**, Equation 4) [39]. Quantum yields of slightly greater than unity for the addition of **R^∙^** to methyl vinyl ketone lend support to chain processes in which BNA**^∙^** (**8**) can also transfer an electron to **6** [39]. Thin film deposition (Equation 5) could then occur via grafting of radicals to generate Au–C bonds at the surface [3–5,21–28], adsorption of RH to the surface, and crosslinking of radicals at the surface [42–44].

While this approach to alkyl radical formation has been exploited in the synthesis of small organic molecules [35–40], it has not been used for the deposition of thin films. Benefits of such an approach include mild visible-light irradiation with safe and inexpensive light sources, use of phthalimide ester precursors **6** with high functional group tolerance, and avoidance of radical oxidation under reducing conditions (i.e., with benzyl nicotinamide).

We also combined thin film deposition with particle lithography [10,17,45–49]. This approach to surface patterning uses photomasks consisting of silicon dioxide mesospheres (*d* = 500 nm) on surfaces to protect small, discrete regions of the surface from the photosensitized thin film deposition. Subsequent removal of the mesospheres with ultrasonication in ethanol results in the formation of nanopores with exposed substrate at the bottom. We have previously demonstrated the utility of nanopores for the measurement of film thickness using AFM cursor profiles and for a head-to-head comparison of the chemical and mechanical stability of a polynitrophenylene multilayer to a thiol thin film on Au(111) [17].

In the work described herein, visible-light-photosensitized deposition of alkyl radicals was combined with particle lithography to generate strongly attached alkyl thin films on Au(111). Further, AFM nanoshaving [50] experiments performed at forces as high as 10 nN showed that removal of a decyl radical-derived film using mechanical force could not be effected easily. Photosensitizer (Ru(bpy)_3_Cl_2_), phthalimide ester, BNAH, and blue LED irradiation all proved necessary for the deposition of robust films that could survive nanoshaving. In addition to characterizations with AFM, grazing angle infrared reflectance–absorbance spectroscopy (IRRAS), X-ray photoelectron spectroscopy (XPS), time-of-flight secondary ion mass spectrometry (TOF SIMS), and contact angle goniometry provided additional information about the structure and composition of the films.

## Experimental

**Materials and reagents.** 11-Aminoundecanoic acid (97%), *N*-hydroxyphthalimide (97%), *N,N’*-dicyclohexylcarbodiimide (99%), 4-(dimethylamino)pyridine (99%, Reagent Plus), di-*tert*-butyl dicarbonate (99%, Reagent Plus), 4 Å molecular sieves, ethanol (99.5%), and Ru(bpy)_3_Cl_2_ (99.95%) were purchased from Sigma-Aldrich and used without further purification. Undecanoic acid (98%) and 1-benzyl-1,4-dihydronicotinamide (BNAH, 95%) were purchased from TCI and used without further purification. ^1^H and ^13^C NMR spectroscopy was performed on a Bruker AV-400 spectrometer. Glassware was flame-dried under vacuum and backfilled with dry nitrogen prior to use. Acetonitrile for thin film deposition procedures was purified according to the method published by Pangborn et al. [51]. Deuterated solvents were purchased from Cambridge Isotope Labs. The irradiation source was two 4 W sapphire blue LED flex strips from Creative Lighting Solutions (Cleveland, OH, USA) wrapped around a crystallizing dish.

**Preparation of gold surfaces with a mesosphere mask.** Template-stripped gold substrates were prepared by a previously reported procedure [52]. Glass discs were glued to freshly prepared gold films using an epoxy (EPO-TEK, Billerica, MA) and cured at 150 °C for 1–2 h. The glass discs (12 mm diameter, purchased from Ted Pella, Inc.) were stripped from mica using a pair of tweezers to expose a clean, atomically flat Au(111) surface. Residual mica was removed from the Au surface by briefly soaking in THF. Size-sorted silica mesospheres with an average diameter of 500 nm (Thermo Scientific) were cleaned by centrifugation and suspension in water (three cleaning cycles). A 10 µL drop of the silica mesosphere suspension was placed onto the template-stripped gold substrates, dried in air for 2 h, and then oven-dried for at least 96 h. The final heating step was used to temporarily anneal the silica spheres to the substrate to prevent displacement during immersion in solutions. After completion of the procedures, removal of the surface mask was accomplished by sonication in ethanol.

**Preparation of phthalimide ester compounds.** Phthalimide esters were synthesized according to a known procedure [38] and purified with silica gel chromatography. NMR and high-resolution mass spec. data for the new compounds Phth–Me and Phth–NHBoc appear in the Supporting Information File 1.

**Procedure for preparation of thin films for AFM analysis.** Acetonitrile (MeCN) from a solvent purification system [51] was dried over 4 Å molecular sieves for at least 24 h. Phthalimide ester (0.2 mmol), BNAH (0.2 mmol), and Ru(bpy)_3_Cl_2_ (0.0056 mmol) were added to a 50 mL Erlenmeyer flask charged with a stir bar. The Au(111) substrate with mesospheres was then added. The flask was then sealed with a rubber septum and a dry N_2_ line was introduced. MeCN (2 mL) was added and the reaction mixture was stirred until only the Ru(bpy)_3_Cl_2_ remained undissolved (Ru(bpy)_3_Cl_2_ remained undissolved until irradiation). Stirring was performed carefully so as to avoid contact between the Au(111) substrate and the stirbar. The reaction mixture was then irradiated with blue LEDs for 30 min (see Supporting Information File 1 for a photo). Upon completion of irradiation, the reaction mixture was decanted. The Au(111) substrate was washed twice with 2 mL deionized water and twice with 2 mL 99.5% ethanol. The substrate was then ultrasonicated for 2 min in 99.5% ethanol followed by ultrasonication for 2 min in deionized water before imaging. These samples were also analyzed with XPS and TOF SIMS. For the deposition of Phth–Me-derived films (vide infra) under standard conditions (phthalimide ester, Ru(bpy)_3_Cl_2_, BNAH, blue LED irradiation), samples were prepared in triplicate and the AFM data is representative. All controls (without Ru(bpy)_3_Cl_2_, without BNAH, without blue LED irradiation) were duplicated. AFM data shown here and in the Supporting Information File 1 are representative.

**Procedure for preparation of samples for IRRAS and contact angle goniometry.** Polycrystalline 100 nm Au-film-coated glass slides obtained from Platypus Technologies (catalog number Au.1000.SL1) were cut into sections of approximately 1 × 1 inch and cleaned using UV/ozone for 24 h. To a 125 mL Erlenmeyer flask charged with a stirbar was added phthalimide ester (0.4 mmol), BNAH (0.4 mmol), and Ru(bpy)_3_Cl_2_ (0.0056 mmol). The freshly cleaned Au film-coated glass was then added. The flask was then sealed with a rubber septum and a dry N_2_ line was introduced. MeCN (4 mL) was added and the reaction mixture was stirred until only the Ru(bpy)_3_Cl_2_ remained undissolved. Stirring was performed carefully so as to avoid contact between the Au substrate and the stirbar. The reaction mixture was then irradiated with blue LEDs for 3 h (see Supporting Information File 1 for photo). After irradiation, the reaction mixture was decanted. The Au substrate was washed with 10 mL deionized water and 10 mL of 99.5% ethanol before analysis. Duplicate samples were prepared for the deposition of films using Phth–Me and Phth–NHBoc. IRRAS/contact angle data are representative.

**Atomic force microscopy (AFM):** Model 5500 and 5420 scanning probe microscopes (Keysight Technologies, Santa Rosa, CA) were used to characterize samples. Images were collected with Pico View v 1.12 software. Tips with an average spring constant of 40 N/m (Budget Sensors, Innovative Solutions Bulgaria Ltd.) were used to acquire topography and corresponding phase images with tapping mode. Nanoshaving experiments were conducted using a liquid cell containing ethanolic solution. Contact mode in liquid was used for nanoshaving using tips with an average spring constant of 0.6 N/m (Bruker Instruments, Camarillo, CA, USA). Digital images were processed with Gwyddion (v 2.30) software [53].

**Grazing angle infrared reflectance absorbance spectroscopy (IRRAS):** IRRAS spectra were recorded using a Bruker Tensor 27 instrument equipped with a liquid N_2_-cooled MCT probe and an 80Spec attachment from Pike Technologies. The angle of incidence was 80°. For each sample, 2056 scans were accumulated with a spectral resolution of 4 cm^−1^.

**Contact angle goniometry (water contact angles):** Water contact angles were measured with a VCA Optima instrument (AST Products, Inc.). Two µL of deionized water were deposited on the top of each sample in the horizontal position. At least six measurements were calculated for each sample using VCA Optima software.

**X-ray photoelectron spectroscopy (XPS):** Photoelectron spectra were obtained using a PHI 5000 VersaProbe Scanning ESCA Microprobe (Physical Electronics, Chanhassen, MN) and a Perkin Elmer 5000 ESCA system each equipped with a monochromatic Al Kα X-ray source (*E*_p_ = 1486.7 eV). Typically, the pressures of the chambers were <7 × 10^−10^ mbar during analysis. The XPS spectra were measured with a pass energy of 23.5 eV and energy step 0.125 eV, and collected at 45° to the normal of the sample surface. The binding energies (*E*_B_) were calibrated using the Au 4f_7/2_ photoelectron peak (*E*_B_ = 84.00 eV).

**Time-of-flight secondary ion mass spectrometry (TOF SIMS):** Time-of-flight secondary ion mass spectra were measured using an ION TOF IV spectrometer (ION TOF Inc., Chestnut Hill, NY) equipped with a Bi liquid metal ion gun. Briefly, the spectrometer consists of three vacuum chambers each separated by a gate valve. Samples are introduced via a loadlock. The preparation and analysis chambers are maintained at ≤7 × 10^−9^ mbar. The primary Bi^+^ ions had a kinetic energy of 25 keV, were contained within a ≈ 100 nm diameter probe beam, and were rastered over a (100 × 100) μm^2^ area. All spectra were obtained in the static regime using a total ion dose of less than 10^10^ ions·cm^−2^. The secondary ions were extracted into a time-of-flight mass spectrometer using a potential of 2 kV and reaccelerated to a kinetic energy of 10 keV before arriving at the detector. At least three areas were examined for each sample, and the reported spectra are representative of the data obtained.

## Results and Discussion

**Scheme 2:**
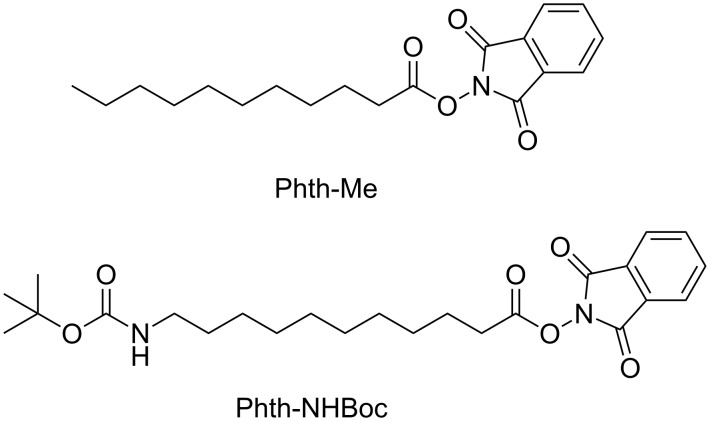
Phthalimide esters used for the deposition of thin films.

For the purposes of this study, we synthesized two phthalimide esters (Scheme 2). We will use the abbreviations “Phth–Me” (methyl-terminated) and “Phth–NHBoc” (NHCO_2_*t*Bu-terminated) to refer to the phthalimide esters depicted in Scheme 2 while the terms “Au–Me” and “Au–NHBoc,” will be used to describe films deposited on Au using the respective phthalimide esters.

To accomplish thin film growth, Au(111) surfaces masked with mesospheres were immersed in solutions of phthalimide ester, benzyl nicotinamide, and the photosensitizer Ru(bpy)_3_Cl_2_ in CH_3_CN and irradiated for 30 min before rinsing/ultrasonication procedures. The resulting thin films on Au were subjected to AFM, XPS, and TOF SIMS analysis. Alternatively, glass-backed slides consisting of polycrystalline Au were irradiated in the presence of phthalimide ester, benzyl nicotinamide and Ru(bpy)_3_Cl_2_ for a duration of 3 h. The resulting thin films were subjected to contact angle goniometry and IRRAS analysis.

**Figure 1:**
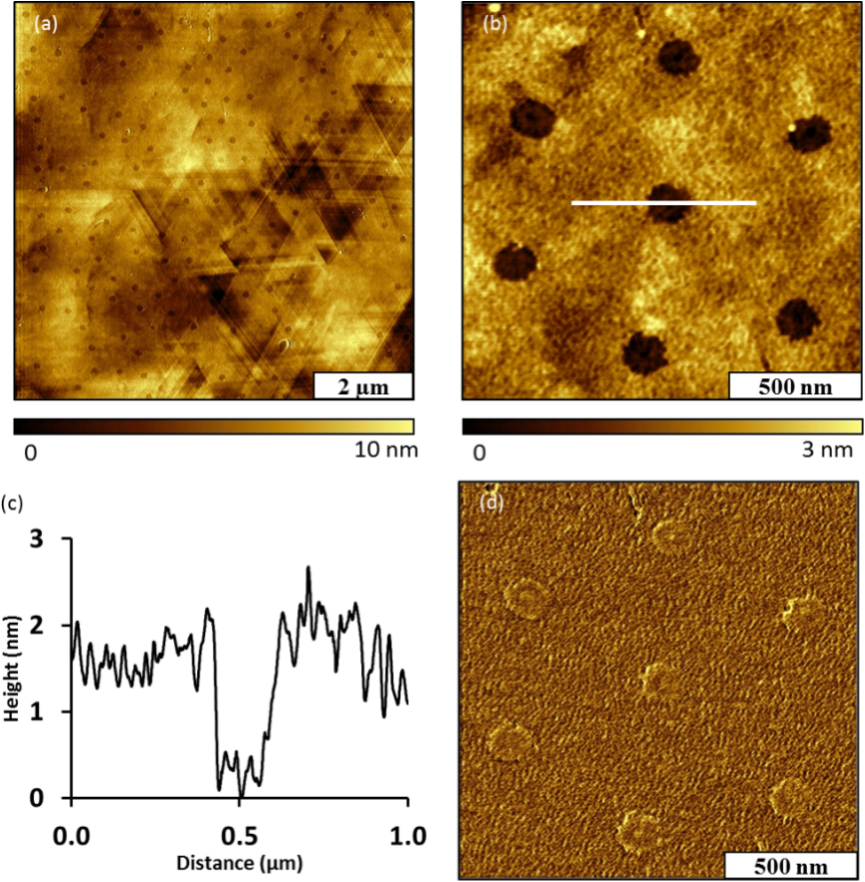
Nanopores within a film of Au–Me on Au(111) produced using immersion particle lithography and the radical precursor Phth–Me. (a) Topography image acquired in air; (b) zoom-in view of hexagonally packed nanopores; (c) cursor profile across a single nanopore in (b); (d) corresponding phase image of (b).

**Atomic force microscopy (AFM):** The deposition of a thin film using phthalimide ester Phth–Me combined with particle lithography produced a periodic arrangement of nanopores within a thin film of Au–Me on Au(111). The sample was imaged with tapping mode in ambient conditions. The sites of nanopores of uncovered gold substrate that were protected by the mesosphere mask appear as dark circles (Figure 1a). The characteristic features of a gold surface such as triangular terrace steps and flat domains are apparent in the topography image. The triangular facets result from the orientation of Au(111) on mica [54]. The surface coverage of the Au–Me thin film measured 79% for an array of nanopores with 500 nm periodicity. A hexagonal arrangement of seven nanopores within the film is shown in closer detail in Figure 1b. The edges of the nanopores are distinct and well defined to form a circular geometry. The distance between adjacent nanopores corresponds to the 500 nm diameter of the silica particles used with particle lithography. The film thickness measured 1.5 ± 0.2 nm from the depth of the nanopores, and the diameter measured 150 ± 8 nm. The lateral dimensions of the nanopores indicate the actual area of contact between the particles of the surface mask and the gold substrate, which is quite a bit smaller than the periodicity. A representative cursor profile of a nanopore from the zoom-in topograph is shown in Figure 1c. A photografted alkyl radical contains a 10 carbon chain which has a theoretical chain length of 1.5 nm, however, the observed film thickness is probably coincidental. It is likely that the upright conformations and surface assembly seen with thiol SAMs [55–58] is not operative with our method, and water contact angles support this assertion. Two distinct areas within the imaging frame are revealed in the phase image of Figure 1b indicating the areas of bare gold surrounded by the thin film.

**Figure 2:**
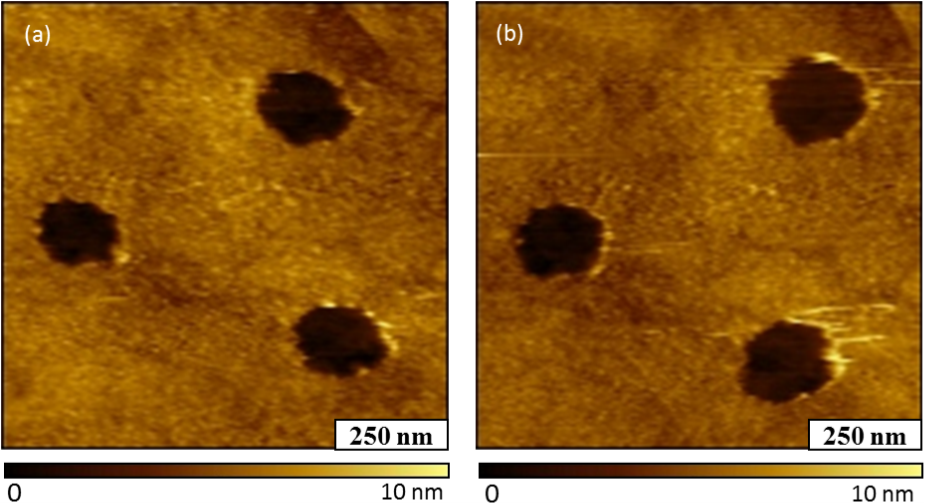
Attempted nanoshaving of the Au–Me Film on Au (111). (a) Topography image of nanopores within the film; (b) topography image acquired after nanoshaving; the film was not displaced.

The robustness of the Au–Me film was evaluated with a nanoshaving experiment conducted in ethanol (Figure 2). In this experiment, a 1 × 1 μm^2^ area with three nanopores within the film was imaged using contact mode (Figure 2a). The nanopores were selected as a reference site for the location and height of the nanoshaved area. Then, the same area was nanoshaved with 10 nN of force on the AFM tip using 10 sweeps. This amount of force has been used successfully in previous experiments for nanoshaving alkanethiol SAMs. For example, one of our laboratories has demonstrated that a series of ω-functionalized alkanethiols could be removed with the application of forces from 2–9 nN using a Si_3_N_4_ AFM tip [59]. The post-nanoshaving film is shown in Figure 2b, and does not reveal any displacement. The nanopores persist with the original shapes and location within the topography frame, indicating that the film was not removed by the scratching action of the AFM tip. At the right edges of nanopores there are a few line patterns showing a few loose adsorbate molecules that were dragged along the direction of the AFM tip movement. Since the patterns appear at the edges of the nanopores, most likely these were ‘piled’ around the edges of the nanopores of Figure 2a. Since the Au–Me film could not be readily removed by nanoshaving, the film is robust possibly due to strong attachment or crosslinking.

**Figure 3:**
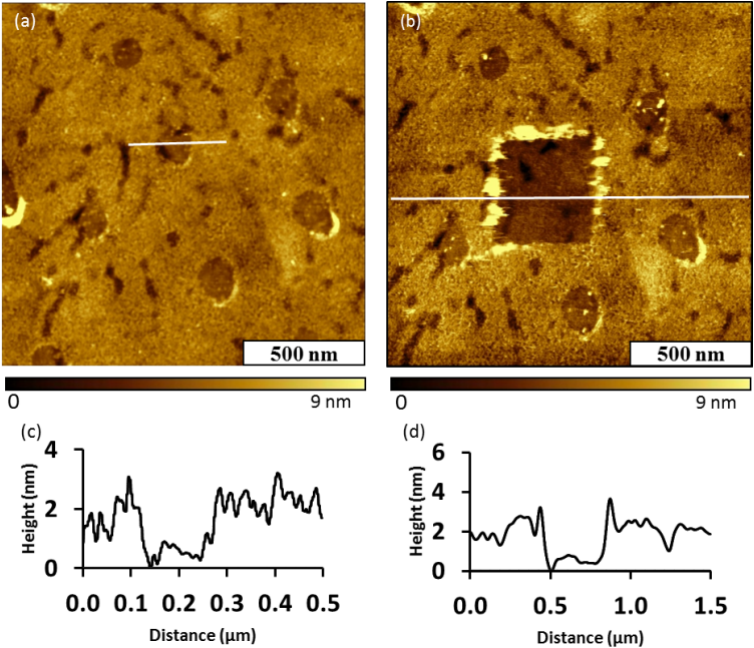
Au–Me thin film prepared in the absence of Ru(bpy)_3_Cl_2_ was not robust. (a) Topography image of nanopores within the film; (b) topograph of a nanoshaved rectangular area; (c) cursor profile of an individual nanopore in (a); (d) cursor profile for the nanoshaved area in (b).

A control experiment was conducted to examine the outcome of thin film deposition in the absence of the photosensitizer Ru(bpy)_3_Cl_2_. The sample exhibits spontaneous surface attachment of components of the mixture on gold (Figure 3a). A thin film also formed on the nanopores wherein the mesospheres had been removed. The reasons for this film formation in the nanopores are not clear. The diameter of the nanopores measured 135 ± 16 nm. The nanopores exhibit a hexagonal arrangement with a periodicity of 500 nm that matches the dimensions of the mesospheres used as a surface mask. The robustness of the film was evaluated with nanoshaving experiments conducted in ethanol. A square pattern was nanoshaved within the Au–Me thin film using 10 nN of force applied to the AFM tip for 10 sweeps. Some of the molecules displaced by the action of the AFM tip piled up at the boundaries of the nanoshaved square, however, it appears that the molecules were cleanly removed from the area within the pattern. The thickness of the film measured 1.5 ± 0.2 nm referencing the uncovered area of gold within the square pattern as the baseline (Figure 3c). Since the film could be readily removed by the scratching action of the AFM probe, the sample prepared without a photosensitizer is clearly not as robust as the previous film deposited in the presence of Ru(bpy)_3_Cl_2_ (Figure 2).

We also conducted controls in the absence of irradiation, in the absence of phthalimide ester, and in the absence of BNAH. Loosely attached thin films akin to the one shown in Figure 3 were observed in the absence of irradiation. No nanopores were observed in the absence of either Phth–Me or BNAH, demonstrating that no film growth had taken place (see Supporting Information File 1). All of the components of the procedure (phthalimide ester, Ru(bpy)_3_Cl_2_, BNAH, and blue LED irradiation) are necessary for the formation of robust thin films as determined with nanoshaving.

**Figure 4:**
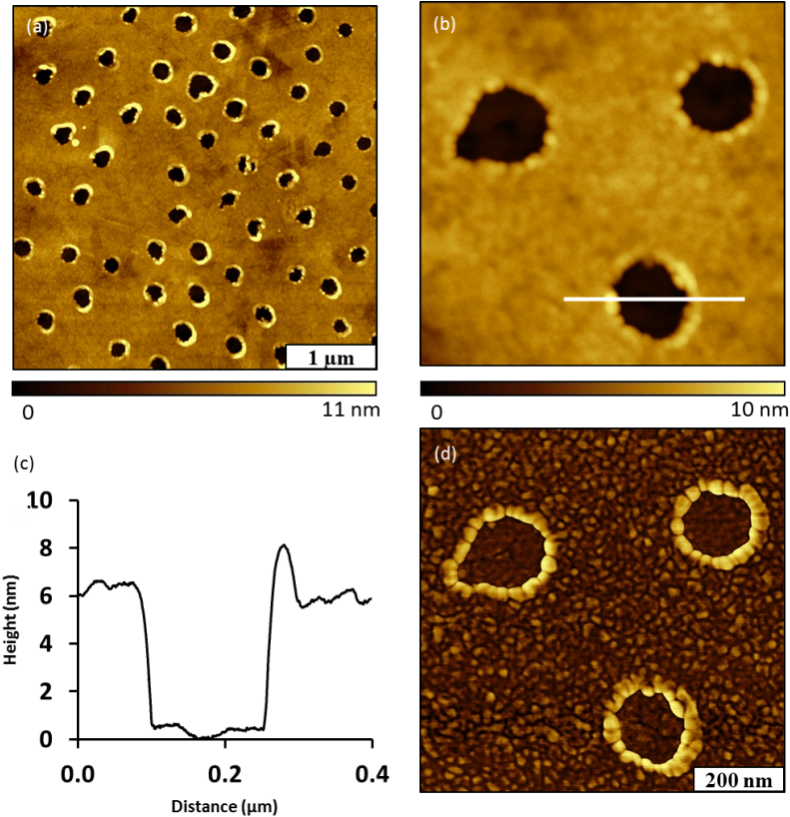
Film of Au–NHBoc prepared on Au(111) with nanopores using particle lithography. (a) Topograph of nanopores; (b) zoom-in topography view of nanopores; (c) cursor profile for the line in (b); (d) corresponding phase image of (b).

The steps of film deposition of phthalimide ester Phth–NHBoc combined with particle lithography also produced a periodic arrangement of nanopores within a thin film of Au–NHBoc on Au(111) (Figure 4). The surface coverage of the Au–NHBoc thin film measured ≈85% for a sample prepared with 500 nm diameter particles (Figure 4a). The final arrangement of the nanopores produced within the films depends on the organization of particles on the surface mask. In this example, there are a few missing or irregularly shaped nanopores that are caused by defects in the arrangement of mesospheres in the initial mask. A high resolution view of three individual nanopores is shown in Figure 4b. A compact film with a relatively smooth morphology is observed in areas between the nanopores. The thickness of the thin film measured 6.0 ± 0.2 nm as shown with a representative cursor profile in Figure 4c. The diameter of the nanopores measured 166 ± 26 nm.

**Figure 5:**
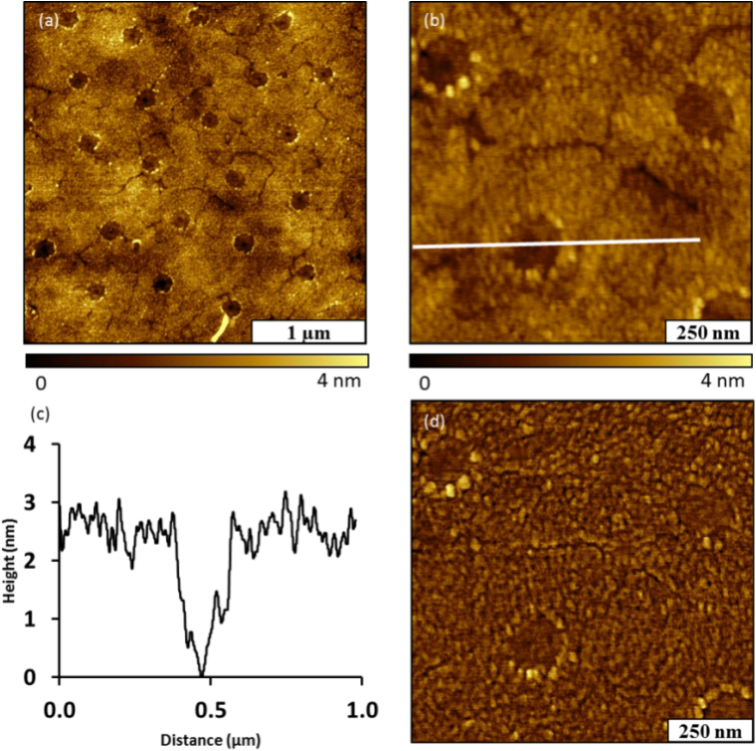
Au–NHBoc film after storage in ambient conditions for six months. (a) Topography image; (b) zoom-in view; (c) cursor profile for the line in (b); (d) corresponding phase image of (b).

The Phth–NHBoc phthalimide ester produces an alkyl radical that has a terminal *tert*-butyl group and chain length of ≈1.8 nm. The measured thickness of the Phth–NHBoc film necessitates a multilayer formed during the reaction. Formation of multilayers, which is likely operative with Au–Me and Au–NHBoc, will be discussed. Reasons for the thickness of the Au–NHBoc thin film relative to that of Au–Me deposited under otherwise identical conditions still require determination. However, it is reasonable to assume that the small, non-polar methyl group at the terminus of the decyl radical will have a profoundly different influence at various stages of Au–Me film growth than the relatively large and polar –NHCO_2_*t*Bu group of the *tert*-butoxycarbonylaminodecyl radical during various stages of Au–NHBoc film growth. These differing influences could result in the dramatically different film thicknesses that are observed. The morphology of the Au–NHBoc film was examined, after storing a sample in ambient conditions for six months, using tapping mode AFM (Figure 5). The arrangement and locations of nanopores can still be resolved with AFM topographs (Figure 5a). The surface coverage of the Au–NHBoc film measured 83%, which is consistent with the value measured from the freshly prepared sample in Figure 4. The thickness of the film decreased with time to 2.5 ± 0.2 nm (Figure 5c). The diameter of the nanopores measured 159 ± 11 nm, which closely matches the dimensions of the fresh sample. The boundaries of the nanopores can be discerned in the corresponding phase image of Figure 5d. Since the film coverage remained the same over a period of 6 months, this suggests that the film is highly robust.

**Contact angle goniometry.** The wettability of both Au–Me and Au–NHBoc thin films on polycrystalline Au slides was investigated by measuring the water contact angles of freshly prepared samples. The average water contact angles were 91 ± 4° (Au–Me) and 93 ± 3° (Au–NHBoc), demonstrating that the surfaces are hydrophobic. The contact angles measured for Au–Me are substantially smaller than the value measured for decanethiol SAMs on Au (109 ± 2°) [60] wherein the water droplet is largely in contact with the terminal methyl groups of the crystalline film. This suggests that Au–Me and Au–NHBoc films in this study are relatively disordered and that water droplets are in contact with the methylene-rich chains derived from Phth–Me and Phth–NHBoc. Indeed, the water contact angle measured on low-density polyethylene is a similar 94° [61].

**Figure 6:**
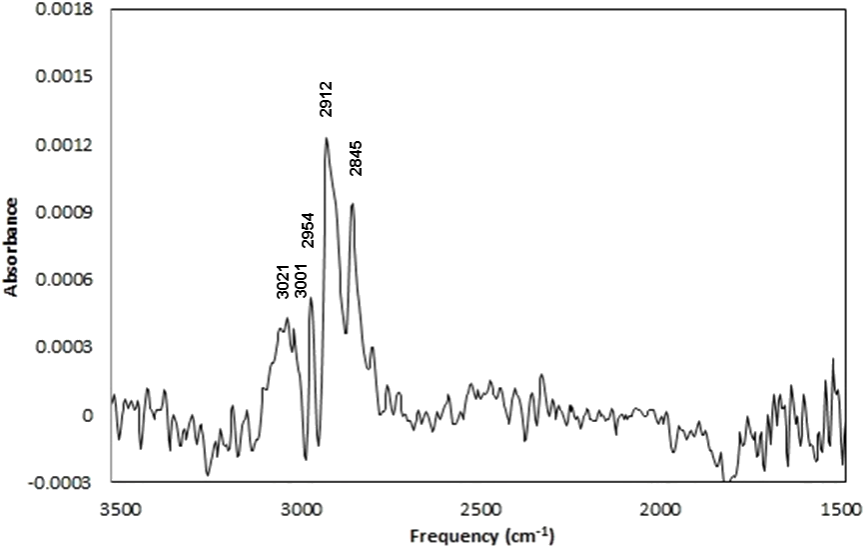
IRRAS Spectrum of Au–Me.

**Figure 7:**
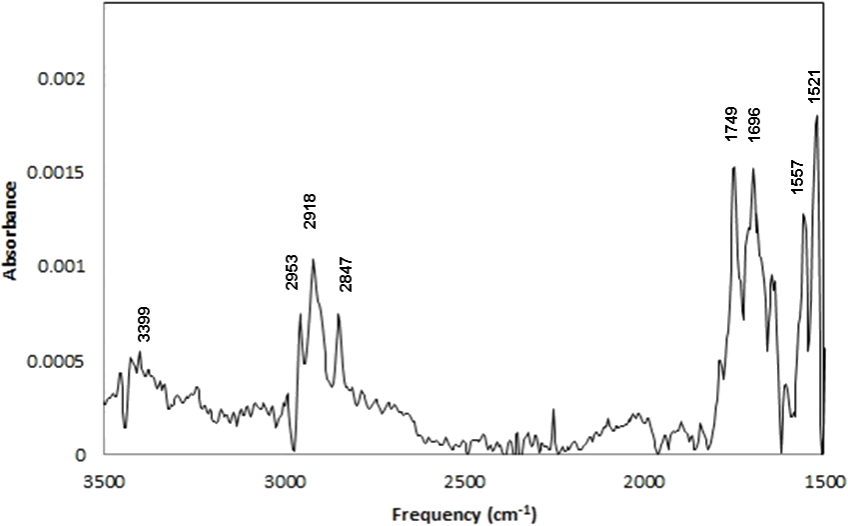
IRRAS Spectrum of Au–NHBoc.

**IRRAS.** IR spectra of Au–Me and Au–NHBoc films are depicted in Figure 6 and Figure 7 (respectively) while some of the tabulated data are depicted in Table 1. The IR spectrum of Au–Me (Figure 6) displays C–H stretching at 2954 cm^−1^, 2912 cm^−1^, and 2845 cm^−1^ (assigned to CH_3_ asymmetric, CH_2_ antisymmetric, and CH_2_ symmetric stretching based on previous reports) [62–63]. In addition, signals at 3021 and 3001 cm^−1^ suggest the presence of alkenes in the film. Noteworthy is the absence of any significant signals in the carbonyl region. The IR spectrum of the precursor Phth–Me displays carbonyl stretching at 1742, 1788, and 1815 cm^−1^ (see Supporting Information File 1), characteristic of phthalimide ester carbonyl groups [64]. The adventitious adsorption of unreacted Phth–Me or phthalimide (1730, 1752, and 1775 cm^−1^) [64] is not extensive, and XPS analyses (absence of nitrogen) further corroborate this assertion. Finally, IRRAS spectra with reasonable signal-to-noise ratios for Au–Me films deposited in the absence of Ru(bpy)_3_Cl_2_ could not be recorded after multiple attempts.

**Table 1:** Selected signals detected with IRRAS.

Thin Film	IRRAS Signals (cm^−1^)

Au–Me	3021, 3001, 2954, 2912, 2845
Au–NHBoc	3399, 2953, 2918, 2847, 1749, 1696, 1557, 1521

The IR spectrum of Au–NHBoc (Figure 7) also displays characteristic C–H stretching at 2953, 2918, and 2847 cm^−1^. In addition, we assign N–H stretching to a signal at 3399 cm^−1^. Noteworthy is the presence of the expected *tert*-butoxycarbonyl-associated carbonyl stretching at 1696 cm^−1^. Further, signals at 1557 and 1521 cm^−1^ suggest C–N stretching. As with Au–Me, the absence of carbonyl stretching at 1815 cm^−1^ weighs against the adventitious adsorption of unreacted Phth–NHBoc while the absence of phthalimide is not as easy to confirm. Indeed, the origin of an apparent carbonyl stretch at 1749 cm^−1^ is not clear, but this indicates that some phthalimide may be present in the film. Notably absent with the Au–NHBoc film are the signals at 3001 and 3021 cm^−1^ that are seen in the Au–Me film.

**Figure 8:**
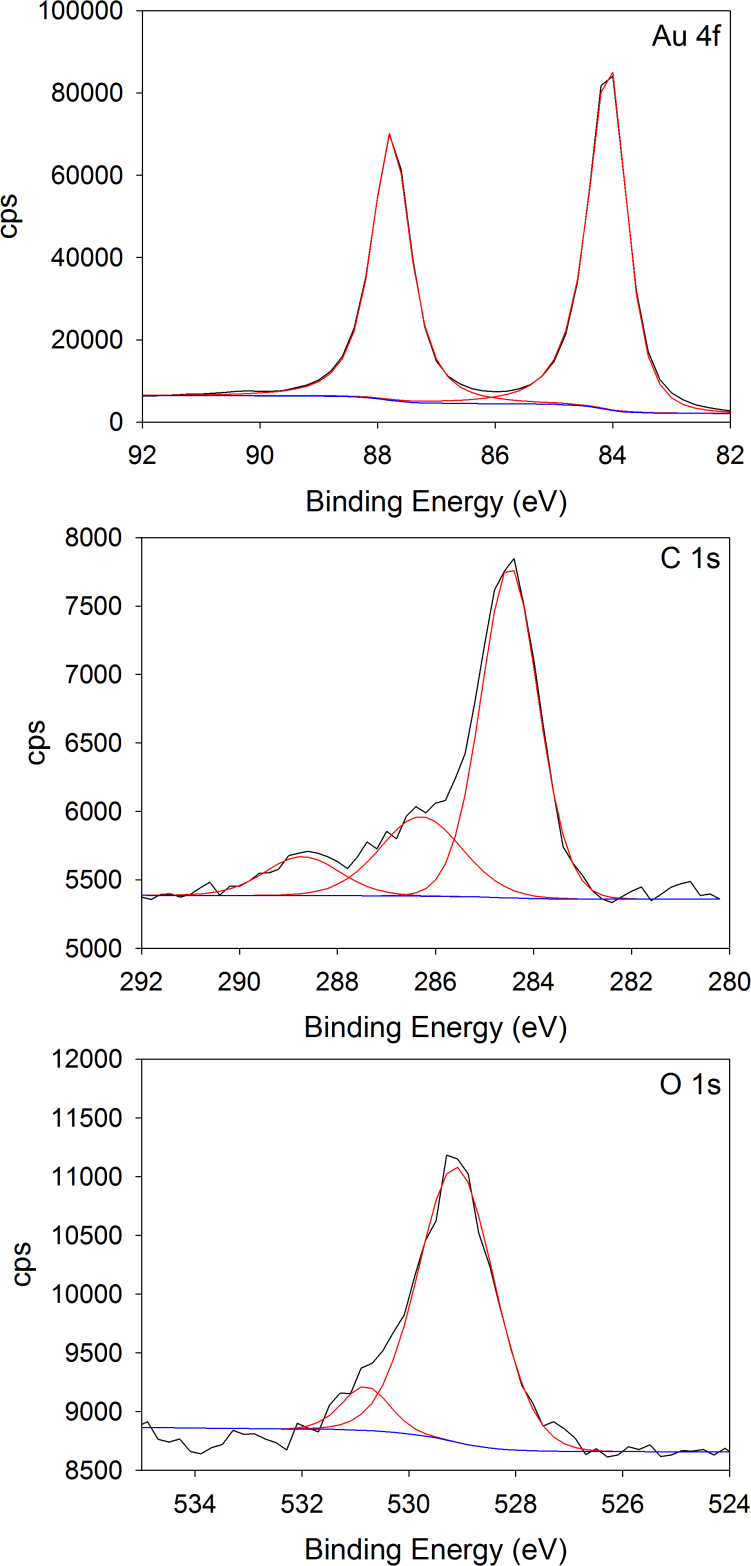
Au 4f, C 1s and O 1s photoelectron spectra of the deposited Au–Me layer.

**XPS.** The XPS data indicate that decyl radicals were deposited onto the gold surface with Au–Me samples (Figure 8). We observe only Au, O and C present in the XPS spectra of Au–Me (see Supporting Information File 1). The absence of N suggests that any adventitious deposition of intact Phth–Me, phthalimide, BNAH, or their derivatives is minimal (see Supporting Information File 1). With the film deposited in the presence of Ru(bpy)_3_Cl_2_, BNAH, and Phth–Me, the presence of the Au 4f_7/2_ and Au 4f_5/2_ photoelectron peaks indicates that the layer is relatively thin which is consistent with the thickness of 1.5 ± 0.2 nm measured by AFM. The C 1s spectrum can be fitted to three contributions (Figure 8, observed at 284.6 eV, 286.5 eV and ≈289 eV) which are assigned to –*C*H_2_–, –*C*O– and –*C*O_2_, respectively [65]. Notably absent are any contributions at <284 eV [3,24], suggesting that formation of metal–carbon bonds at the surface is not extensive. The O 1s spectrum is relatively broad and the asymmetric binding energy envelope can be fitted to two contributions at 529.3 eV and ≈531 eV (Figure 8). The lower binding energy is consistent with adventitious oxygen or with the formation of gold oxides (Au_2_O_3_, *E*_B_ = 530.2 eV) [65–66], but we note that there is no evidence of Au oxidation (Au_2_O_3_, *E*_B_(Au 4f_7/2_) = 85.9 eV) [65]. The higher binding energy can be assigned either to the formation of metal carbonates [65] or C=O bonds [65,67]. Taken together, the C 1s, O 1s, and Au 4f data indicate that the gold substrate is not oxidized.

The data are also consistent with the AFM results indicating that [Ru(bpy)_3_]^2+^ is required for the formation of robust, adhesive Au–Me films. In the absence of [Ru(bpy)_3_]^2+^, the XPS data indicate that the deposited layer has a similar chemistry to that of the Au–Me film deposited in the presence of [Ru(bpy)_3_]^2+^ (see spectra in Supporting Information File 1). The C 1s spectrum can be fitted to three contributions at 284.6 eV, 286.5 eV, and ≈289 eV, respectively, which are assigned to –*C*H_2_–, –*C*O–, and –*C*O_2_, respectively [65]. However, in the O 1s spectrum, fitting results in two peaks that are observed at higher binding energies (≈533.2 eV and 531.8 eV). These binding energies are consistent with oxygen present in higher nominal oxidation states such as those found in organic molecules including polymers [65], suggesting that the deposited layer does not strongly adhere to the substrate.

**Figure 9:**
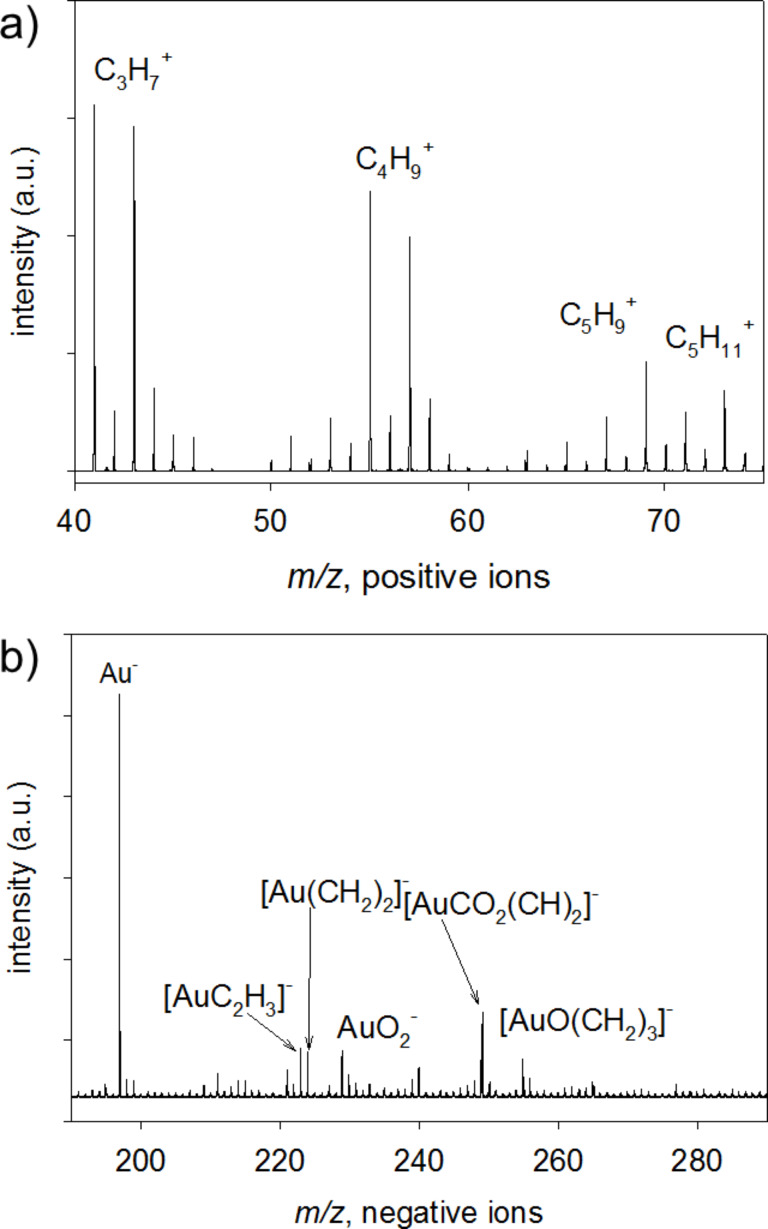
a) Positive ion mass spectrum *m*/z 40–75 and b) negative ion mass spectrum *m*/*z* 190–290 of the deposited layer (Au–Me).

**TOF SIMS.** In the positive ion mass spectra of Au–Me, ions of the form C_x_H_y_^+^ are observed, indicating that decyl-chains have been deposited onto the gold surface (Figure 9a). In the negative ion spectra, ions of the form [Au(CH_x_)_y_]^−^, [AuCO_2_(CH_x_)_y_]^−^ and [AuCO(CH_x_)_y_]^−^ are observed (Figure 9b). The data indicate that some of the layer may be bound to the Au substrate via Au–C bonds ([Au(CH_x_)_y_]^−^). In light of the XPS data in which metal–carbon bonds were not observed, the number of Au–C bonds would have to be small relative to the number of decyl radicals or decane molecules deposited in the film. The presence of the oxygen-containing ions also indicates that some of the deposited layer may be bound to the substrate via Au–carboxylate (CO_2_–Au) bonds [67–68] which have also been observed in the formation of alkyne monolayers on gold [69]. As before, the lack of Au oxidation observed with XPS indicates that CO_2_–Au bonding at the surface is not likely to be extensive.

**Scheme 3:**
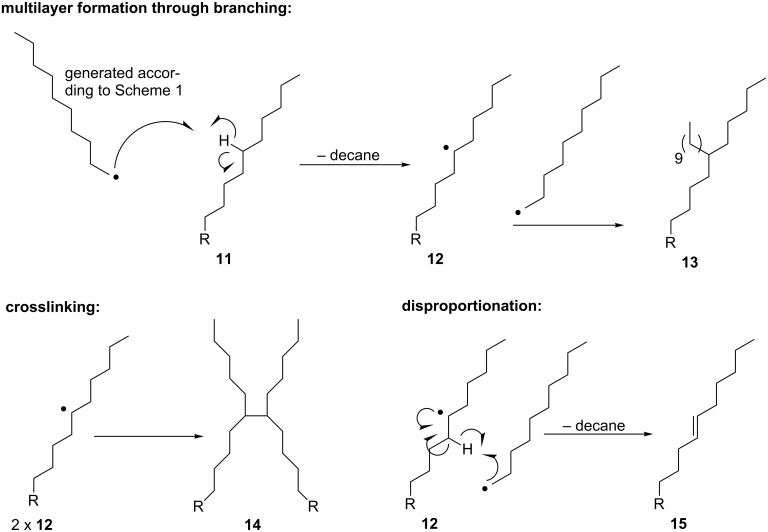
A mechanistic hypothesis for multilayer formation, crosslinking, and alkene formation.

**Discussion.** Taken together, data acquired using various modes of AFM imaging and nanoshaving, contact angle goniometry, IRRAS, XPS and TOF SIMS indicate that the visible-light-promoted formation of alkyl radicals using phthalimide esters, Ru(bpy)_3_Cl_2_, BNAH, and blue LED irradiation in the presence of Au (111) surfaces results in stable but disordered thin films. While the origin of this robust nature is not entirely clear, a number of possibilities exist. Crosslinking of loosely attached spin-cast alkane films to generate mechanically robust films has been induced by the formation of carbon-centered radicals upon bombardment with hydrogen atoms [42] and protons [43] while the crosslinking of liquid alkanes has been demonstrated with radical initiators [44]. Therefore, crosslinking is a possible underlying reason for robustness (see proposed mechanism, Scheme 3). In addition, formation of Au–C and CO_2_–Au bonds at the surface is evidenced by TOF-SIMS but not by XPS, suggesting that limited anchoring of the film may be occurring through covalent bonding to the substrate. Such considerations will be the topics of ongoing investigation.

It is intriguing that loosely attached films are deposited in the absence of Ru(bpy)_3_Cl_2_ and that, further, the chemistry of these films is similar to those deposited in the presence of Ru(bpy)_3_Cl_2_. While this film deposition is poorly understood, a combination of the physisorption of molecules and processes owing to radicals generated through uncatalyzed processes may explain this outcome. In particular, Overman and co-workers have demonstrated that visible-light irradiation of solutions of phthalimide ester and BNAH results in the formation of radicals albeit with a dramatic reduction in efficiency compared to the same reactions performed in the presence of [Ru(bpy)_3_]^2+^ [37]. The mechanism of such processes may involve the intermediacy of charge transfer complexes between phthalimide esters and BNAH [37]. The mechanism for the formation of loosely attached thin films in the absence of blue LED irradiation is also cryptic but may likewise involve inefficient formation of radicals.

Based on observations made with contact angle goniometry, we stated earlier that Au–Me and Au–NHBoc films are disordered. The assertions that these films are disordered (thus, a crystalline, SAM-like film wherein hydrocarbon chains adopt an all-anti conformation is not present) and also have a thickness similar to or greater than the length of decyl and *N*-*tert*-butoxycarbonylaminodecyl radicals in the all-anti conformation can be reconciled by the formation of multilayers in both cases (Au–Me and Au–NHBoc). A mechanism for such multilayer formation has been previously suggested [5], and we elaborate on this with a more detailed mechanistic proposal (Scheme 3) that also accounts for crosslinking. Decyl radicals generated under photochemical conditions (see Scheme 1, Equation 2) can be converted to decane (Scheme 1, Equation 3) or undergo inefficient grafting to the metal surface to generate Au–C bonds. The species **11** (Scheme 3) represents a decyl chain in which R can be H (decane adsorbed to the Au surface), Au (from grafting of a decyl radical to the Au surface) or another alkyl chain (due to crosslinking). Abstraction of a hydrogen atom from **11** by a decyl radical will result in the formation of decane and, most likely, a secondary radical such as **12**. Combination of **12** (which is, once again, either adsorbed or grafted to the Au surface) with another decyl radical will form branched hydrocarbon **13** (Scheme 3). Such processes could serve as a basis for multilayer formation. In addition, crosslinking may occur by combination of radicals **12** (**12**→**14**, Scheme 3) whereas alkene formation may occur through disproportionation (**12**→**15**, Scheme 3) [44]. Au–C and CO_2_–Au bonds in addition to crosslinking would be expected to contribute to the mechanical stability of these films.

Importantly, Scheme 3 is meant to merely provide “snapshots” of multilayer formation through branching, crosslinking through radical-radical coupling, and alkene formation through disproportionation. Scheme 3 does not provide a complete mechanistic hypothesis for photosensitized thin film formation. Indeed, a comprehensive mechanistic hypothesis will require a deeper understanding of the film structure than what has been provided by these seminal studies.

The bond strength of Au–C bonds (in which C is sp^3^-hybridized) at the surface of Au(111) has been estimated at 21.8 kcal/mole whereas the corresponding Au–S bond is 36.8 kcal/mole [70]. Further, the strength of CO_2_–Au bonds at an Au(111) surface is 13.5 kcal/mole [69]. The relatively weak (compared to Au–S) and scarce (detected by TOF SIMS but not XPS) Au–C and CO_2_–Au bonds must therefore provide at best a minor contribution to the overall mechanical stability of Au–Me and Au–NHBoc films relative to other phenomena such as crosslinking. Recall that 9 nN of force applied by an AFM tip readily removed ω-functionalized thiol SAMS from Au [59] whereas 10 nN could not remove Au–Me or Au–NHBoc films.

In considering the process **11**→**12** (Scheme 3), the rate of hydrogen atom abstraction by a primary, carbon-centered radical from a relatively strong bond between an sp^3^-hybridized carbon and hydrogen should be relatively slow. For example, the abstraction of a hydrogen atom by 1-octyl radical from the secondary position of diethyl ether occurs with a bimolecular rate constant of 1.1 × 10^3^ M^−1^·s^−1^ [71]. This is relatively slow for a radical process, and the analogous abstraction of hydrogen from a stronger secondary aliphatic C–H bond in an alkyl chain lacking oxygens should be even slower. We are not aware of any reports on the formation of crosslinked hydrocarbons from linear species when phthalimide ester photosensitization is used for the synthesis of small molecules [35–39]. Nevertheless, formation of the thin film structures proposed herein does not necessarily have to reflect the processes that are occurring the majority of the time in solution (e.g., Equations 3,4, Scheme 1). Events such as the attachment of decyl radical to Au(111), the conversion of **11** to **13**, and the generation of **14** and **15** may be rare, but such rarity should not preclude the formation of a film with nm thickness. Scheme 3 is a working mechanistic hypothesis, and ongoing studies will further elucidate the nature of thin film formation using the reported technique.

## Conclusion

Alkyl thin films were prepared via the generation of alkyl radicals using visible-light irradiation of [Ru(bpy)_3_]^2+^ in the presence of phthalimide esters, BNAH, and submerged Au surfaces. To contrast with previous methods involving the grafting of alkyl halide-derived radicals, this method required visible-light irradiation (with blue LEDs) rather than UV sources. AFM cursor profiles of nanopores derived with particle lithography provided a reliable method for the measurement of film thickness. The deposited alkyl multilayers proved to be robust as determined with AFM nanoshaving experiments and long-term exposure to laboratory air, and this robustness was dependent on the implementation of [Ru(bpy)_3_]^2+^, BNAH, and visible-light irradiation. The robust nature of the alkyl films may be due to crosslinking in addition to limited formation of Au–C and CO_2_–Au bonds at the surface. The films also proved to be disordered as determined with contact angle goniometry. The generation of multilayers is likely a consequence of alkyl groups that undergo a series of radical processes after initial adsorption to or grafting to the Au surface. Thus, C–H abstraction by, and combination with, alkyl radicals from solution (in addition to crosslinking) will result in the formation of branched alkyl chains and multilayer formation. Our ongoing studies will demonstrate, inter alia, the functional group tolerance of this method.

## Supporting Information

File 1 Synthesis and characterization of Phth–Me and Phth–NHBoc, photographs of experimental setups, AFM images of control samples.

## References

[1] Mahouche-Chergui S, Gam-Derouich S, Mangeney C, Chehimi M M (2011). Chem Soc Rev.

[2] Bélanger D, Pinson J (2011). Chem Soc Rev.

[3] Combellas C, Kanoufi F, Osman Z, Pinson J, Adenier A, Hallais F (2011). Electrochim Acta.

[4] Berisha A, Combellas C, Hallais F, Kanoufi F, Pinson J, Podvorica F I (2011). Chem Mater.

[5] Chehimi M M, Hallais G, Matrab T, Pinson J, Podvorica F I (2008). J Phys Chem C.

[6] Pinson J, Podvorica F (2005). Chem Soc Rev.

[7] Allongue P, de Villeneuve C H, Cherouvrier G, Cortès R, Bernard M-C (2003). J Electroanal Chem.

[8] de Villeneuve C H, Pinson J, Bernard M C, Allongue P (1997). J Phys Chem B.

[9] Samanta S, Bakas I, Singh A, Aswal D K, Chehimi M M (2014). Langmuir.

[10] Maldonado S, Smith T J, Williams R D, Morin S, Barton E, Stevenson K J (2006). Langmuir.

[11] Laurentius L, Stoyanov S R, Gusarov S, Kovalenko A, Du R, Lopinski G P, McDermott M T (2011). ACS Nano.

[12] Laforgue A, Addou T, Bélanger D (2005). Langmuir.

[13] Hurley B L, McCreery R L (2004). J Electrochem Soc.

[14] Boukerma K, Chehimi M M, Pinson J, Blomfield C (2003). Langmuir.

[15] Vase K H, Holm A H, Pedersen S U, Daasbjerg K (2005). Langmuir.

[16] Vase K H, Holm A H, Norrman K, Pedersen S U, Daasbjerg K (2007). Langmuir.

[17] Verberne-Sutton S D, Quarels R D, Zhai X, Garno J C, Ragains J R (2014). J Am Chem Soc.

[18] Bouriga M, Chehimi M M, Combellas C, Decorse P, Kanoufi F, Deronzier A, Pinson J (2013). Chem Mater.

[19] Busson M, Berisha A, Combellas C, Kanoufi F, Pinson J (2011). Chem Commun.

[20] Schroll P, Fehl C, Dankesreiter S, König B (2013). Org Biomol Chem.

[21] Berisha A, Combellas C, Kanoufi F, Pinson J, Ustaze S, Podvorica F I (2010). Chem Mater.

[22] Andrieux C P, Gonzalez F, Savéant J-M (1997). J Am Chem Soc.

[23] Brooksby P A, Downard A J, Yu S S C (2005). Langmuir.

[24] Yang F, Hunger R, Roodenko K, Hinrichs K, Rademann K, Rappich J (2009). Langmuir.

[25] Niwa D, Inoue T, Fukunaga H, Akasaka T, Yamada T, Homma T, Osaka T (2004). Chem Lett.

[26] Fellah S, Amiar A, Ozanam F, Chazalviel J-N, Vigneron J, Etcheberry A, Stchakovsky M (2007). J Phys Chem B.

[27] Gros-Jean M, Herino R, Chazalviel J-N, Ozanam F (2000). Mater Sci Eng, B.

[28] Hetemi D, Kanoufi F, Combellas C, Pinson J, Podvorica F I (2014). Langmuir.

[29] Zaera F, Gleason N R, Klingenberg B, Ali A H (1999). J Mol Catal A: Chem.

[30] Lin J-L, Teplyakov A V, Bent B E (1996). J Phys Chem.

[31] Yang M X, Jo S K, Paul A, Avila L, Bent B E, Nishikida K (1995). Surf Sci.

[32] Zaera F, Hoffmann H, Griffiths P R (1990). J Electron Spectrosc Relat Phenom.

[33] Kurita K, Imajo H (1982). J Org Chem.

[34] Barton D H R, Blundell P, Jaszberenyi J C (1989). Tetrahedron Lett.

[35] Tao D J, Slutskyy Y, Overman L E (2016). J Am Chem Soc.

[36] Müller D S, Untiedt N L, Dieskau A P, Lackner G L, Overman L E (2015). J Am Chem Soc.

[37] Pratsch G, Lackner G L, Overman L E (2015). J Org Chem.

[38] Schnermann M J, Overman L E (2012). Angew Chem, Int Ed.

[39] Okada K, Okamoto K, Morita N, Okubo K, Oda M (1991). J Am Chem Soc.

[40] Qin T, Cornella J, Li C, Malins L R, Edwards J T, Kawamura S, Maxwell B D, Eastgate M D, Baran P S (2016). Science.

[41] Prier C K, Rankic D A, MacMillan D W C (2013). Chem Rev.

[42] Liu Y, Yang D Q, Nie H-Y, Lau W M, Yang J (2011). J Chem Phys.

[43] Zheng Z, Xu X, Fan X, Lau W M, Kwok R W M (2004). J Am Chem Soc.

[44] Hulse G E, Kersting R J, Warfel D R (1981). J Polym Sci, Polym Chem Ed.

[45] Nguyen V Q, Sun X, Lafolet F, Audibert J-F, Miomandre F, Lemercier F, Loiseau F, Lacroix J-C (2016). J Am Chem Soc.

[46] Cernat A, Griveau S, Martin P, Lacroix J C, Farcau C, Sandulescu R, Bedioui F (2012). Electrochem Commun.

[47] Corgier B P, Bélanger D (2010). Langmuir.

[48] Santos L, Ghilane J, Lacroix J-C (2012). Electrochem Commun.

[49] Saner C K, Lusker K L, LeJeune Z M, Serem W K, Garno J C (2012). Beilstein J Nanotechnol.

[50] Liu G-Y, Xu S, Qian Y (2000). Acc Chem Res.

[51] Pangborn A B, Giardello M A, Grubbs R H, Rosen R K, Timmers F J (1996). Organometallics.

[52] Wagner P, Zaugg F, Kernen P, Hegner M, Semenza G (1996). J Vac Sci Technol, B.

[53] Nečas D, Klapetek P (2011). Cent Eur J Phys.

[54] Lüssem B, Karthäuser S, Haselier H, Waser R (2005). Appl Surf Sci.

[55] Vericat C, Vela M E, Benitez G, Carro P, Salvarezza R C (2010). Chem Soc Rev.

[56] Love J C, Estroff L A, Kriebel J K, Nuzzo R G, Whitesides G M (2005). Chem Rev.

[57] Schreiber F (2004). J Phys: Condens Matter.

[58] Delamarche E, Bruno M, Biebuyck H A, Gerber C (1996). Adv Mater.

[59] Kelley A T, Ngunjiri J N, Serem W K, Lawrence S O, Yu J-J, Crowe W E, Garno J C (2010). Langmuir.

[60] Mendoza S M, Arfaoui I, Zanarini S, Paolucci F, Rudolf P (2007). Langmuir.

[61] Owens D K, Wendt R C (1969). J Appl Polym Sci.

[62] Snyder R G, Schachtschneider J H (1963). Spectrochim Acta.

[63] Schachtschneider J H, Snyder R G (1963). Spectrochim Acta.

[64] Terent’ev A O, Krylov I B, Timofeev V P, Starikova Z A, Merkulova V M, Ilovaisky A I, Nikishin G I (2013). Adv Synth Catal.

[R65] 65NIST X-ray Spectroscopy Database, Version 4.1; National Institute of Standards and Technology: Gaithersburg, MD, U.S.A., **2012**.

[66] Pireaux J J, Liehr M, Thiry P A, Delrue J P, Caudano R (1984). Surf Sci.

[67] Hooper A, Fisher G L, Konstadinidis K, Jung D, Nguyen H, Opila R, Collins R W, Winograd N, Allara D L (1999). J Am Chem Soc.

[68] Fisher G L, Hooper A E, Opila R L, Allara D L, Winograd N (2000). J Phys Chem B.

[69] McDonagh A M, Zareie H M, Ford M J, Barton C S, Ginic-Markovic M, Matisons J G (2007). J Am Chem Soc.

[70] de la Llave E, Ricci A, Calvo E J, Scherlis D A (2008). J Phys Chem C.

[71] Newcomb M, Kaplan J (1988). Tetrahedron Lett.

