# Supplementary material for: Application of visible-light photosensitization to form alkyl-radical-derived thin films on gold
Source: Beilstein J Nanotechnol. 2017 Sep 6;8:1863–77. doi: 10.3762/bjnano.8.187 (PMC5629420; doi:10.3762/bjnano.8.187)
Supplement: File 1 — Synthesis and characterization of Phth–Me and Phth–NHBoc, photographs of experimental setups, AFM images of control samples. [file Beilstein_J_Nanotechnol-08-1863-s001.pdf]

Supporting Information

for

Application of visible-light photosensitization to form alkyl-radical-derived thin films on gold

Rashanique D. Quarels[1], Xianglin Zhai[1], Neepa Kuruppu[1], Jenny K. Hedlund[2], Ashley A. Ellsworth[2], Amy V. Walker[2,3], Jayne C. Garno[1] and Justin R. Ragains*[1]

Address: [1]Department of Chemistry, Louisiana State University, 232 Choppin Hall, Baton Rouge, LA, 70803, USA, [2]Department of Chemistry and Biochemistry, University of Texas at Dallas, 800 W. Campbell Rd., Richardson, TX, 75080, USA and [3]Department of Materials Science, University of Texas at Dallas, 800 W. Campbell Rd., Richardson, TX, 75080, USA

Email: Justin R. Ragains - jragains@lsu.edu

*Corresponding author

Synthesis and characterization of Phth–Me and Phth–NHBoc, photographs of experimental setups, AFM images of control samples

Table of Contents

**Synthesis of Phth-Me/Phth-NHBoc/NMR spectra**	**S3-S8**
**Photographs of Experimental Setup**	**S9-S10**
**AFM Images of Controls**	**S11-S12**
**FTIR Spectra of Phth-Me/Phth-NHBoc**	**S13-S14**
**XPS Spectra of Controls**	**S15-S16**
**References**	**S17**

Synthesis of undecanoic acid phthalimide ester (Phth-Me):

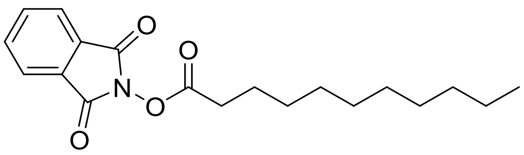

This compound was prepared according to a procedure similar to that reported in [1]. To a 25 °C solution of undecanoic acid (2.00 g, 10.7 mmol) in THF (40 mL) was added *N*-hydroxyphthalimide (2.98 g, 18.3 mmol), 4-dimethylaminopyridine (0.0656 g, 0.537 mmol), and *N,N'*-dicyclohexylcarbodiimide (3.32 g, 16.1 mmol) in succession. After the addition of *N,N'*-dicyclohexylcarbodiimide, the reaction vessel was sealed and a nitrogen line was added. The reaction was magnetically stirred at 25 °C and monitored by TLC until completion. Upon completion, the reaction was filtered using a Buchner funnel. The filter cake was washed with 30 mL CH_2Cl_2. The filtrate was concentrated in vacuo. Column chromatography using 10% ethyl acetate in hexane afforded 2.54 g (71%) of a white solid. ^{1}H NMR (400MHz, $CDCl_3$): δ 7.88 (dd, 2H, *J* = 5.5, 3.1 Hz), 7.78 (dd, 2H, *J* = 5.5, 3.2 Hz), 2.66 (t, 2H, *J* = 7.5 Hz), 1.78 (m, 2H, *J* = 7.4 Hz), 1.44 (m, 2H, *J* = 5.1 Hz), 1.27 (m, 12H), 0.87 (t, 3H, *J* = 6.7 Hz); ^{13}C NMR (100 MHz, $CDCl_3$): δ 169.9, 162.2, 134.9, 129.2, 124.2, 32.1, 31.2, 29.7, 29.6, 29.5, 29.3, 29.0, 24.9, 22.9, 14.3; HRMS m/z calculated for $C_{19}H_{25}NO_4Na$ $(M+Na)^+$ 354.1676, found 354.1686.

Synthesis of *N-tert*-butoxycarbonyl-11-aminoundecanoic acid (en route to PhthNHBoc):

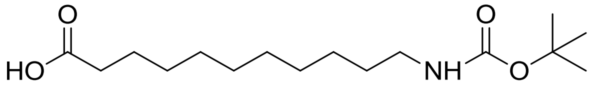

This compound was prepared according to a procedure similar to that reported in [2]. To a suspension of 11-aminoundecanoic acid (1.00 g, 5.0 mmol) in a solution (10.6 mL) of $NaHCO_3$ at half saturation was added sodium carbonate (0.530 g, 5.0 mmol). The resulting suspension was cooled to 0 °C in an ice-water bath. A solution of di-*tert*-butyldicarbonate (1.31 g, 6.0 mmol) in 1,4-dioxane (4.2 mL) was prepared and added to the suspension at once. The reaction mixture was allowed to warm to room temperature and stirred overnight. Upon completion, the stir bar was removed and the reaction

mixture was concentrated in vacuo to remove 1,4-dioxane before being washed with Et_2O (2 × 25 mL) to remove excess di-*tert*-butyldicarbonate. The aqueous layer was treated with cold 1 M HCl dropwise until pH 2. Saturated aqueous NaCl (20 mL) was added to the aqueous layer which was then extracted with 6 x15mL of EtOAC. The combined organic layers were dried over $MgSO_4$, filtered, and concentrated. Column chromatography using 30% ethyl acetate/hexane resulted in the isolation of 1.47 g (98%) of a white solid. The ^{1}H NMR of this compound was consistent with a previous report [3]. ^{1}H NMR (400 MHz, $CDCl_3$): δ 4.52 (bs, 1H), 3.10 (m, 2H), 2.34 (t, 2H, *J* = 7.4 Hz), 1.62 (m, 2H), 1.52 (m, 2H), 1.44 (m, 12H), 1.27 (s, 9H).

Synthesis of *N*-*tert*-butoxycarbonyl-11-aminoundecanoic acid phthalimide ester (PhthNHBoc):

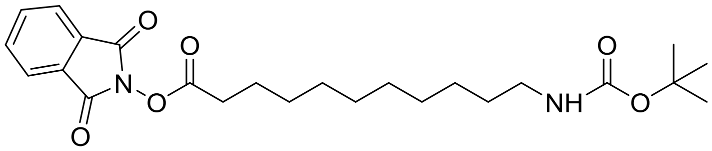

This compound was prepared according to the method used for the synthesis of undecanoic acid phthalimide (Phth-Me) ester starting instead with *N*-*tert*-butoxycarbonyl-11-aminoundecanoic acid (0.682 g, 2.26 mmol). Column chromatography (20% EtOAc in hexanes) resulted in the isolation of 0.911 g (90%) of a white solid. ^{1}H NMR (500 MHz, $CDCl_3$): δ 7.88 (dd, *J* = 5.4, 3.1 Hz, 2H), 7.79 (dd, *J* = 5.5, 3.1 Hz, 2H), 4.50 (s, 1H), 3.11 (m, 2H), 2.66 (t, *J* = 7.5Hz, 2H), 1.78 (p, *J* = 7.5 Hz, 2H), 1.44 (m, 14H), 1.29 (s, 9H); ^{13}C NMR (125 MHz, $CDCl_3$): δ 169.9, 162.2, 156.2, 134.9, 129.2, 124.2,79.2, 40.9, 31.2 30.3, 29.6, 29.49, 29.44, 29.3, 29.0, 28.7, 27.0, 24.9; HRMS *m*/*z* calculated for $C_{24}H_{34}O_6N_2Na$ $(M + Na)^+$ 469.2309, found 469.2318.

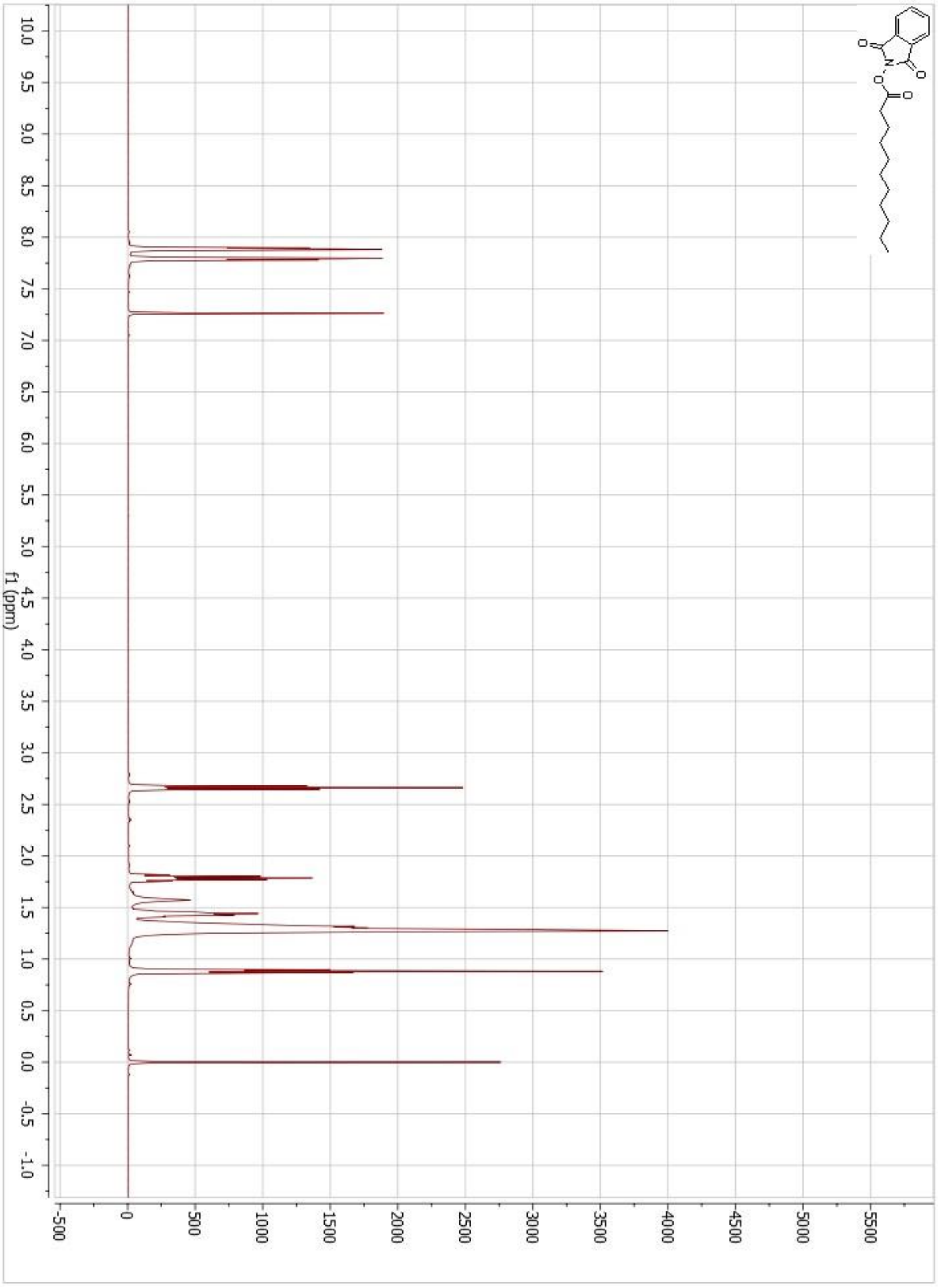

Figure S1: ^{1}H NMR spectrum of undecanoic acid phthalimide ester (Phth-Me).

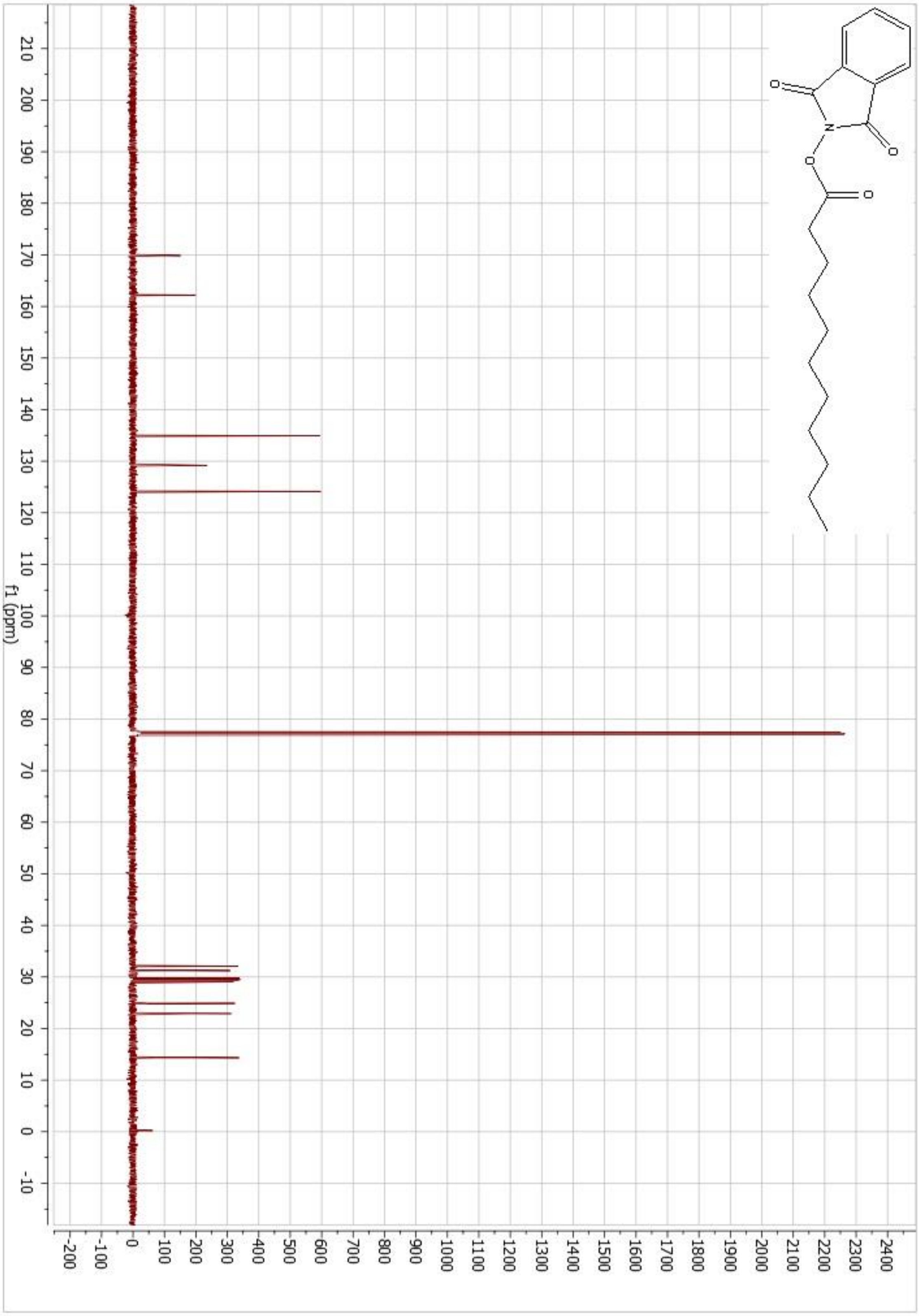

Figure S2: ^{13}C NMR spectrum of undecanoic acid phthalimide ester (Phth-Me).

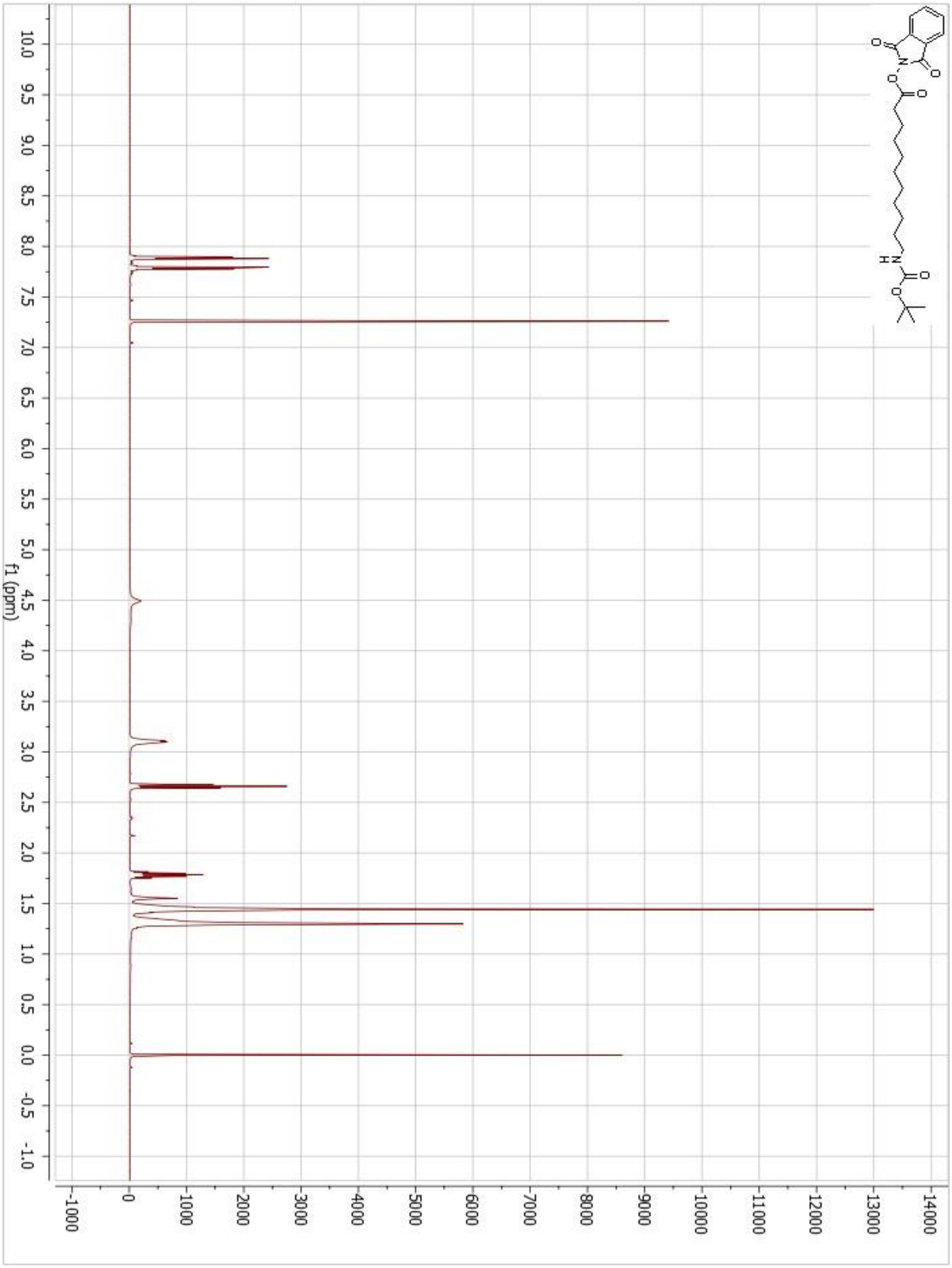

Figure S3: ^{1}H NMR Spectrum of *N-tert*-butoxycarbonyl-11-aminoundecanoic acid phthalimide ester (Phth-NHBoc).

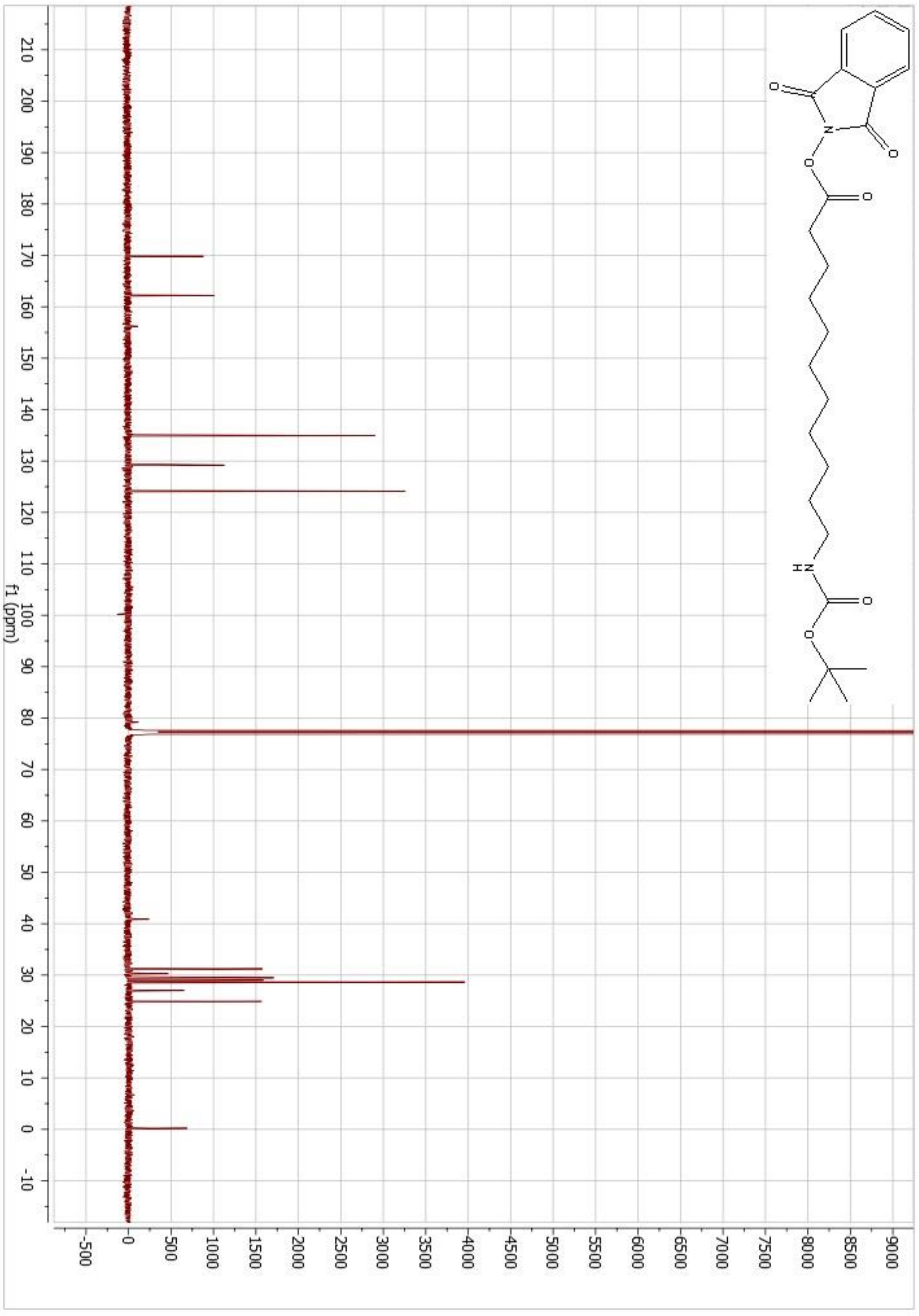

Figure S4: ^{13}C NMR Spectrum of *N*-*tert*-butoxycarbonyl-11-aminoundecanoic acid phthalimide ester (Phth-NHBoc).

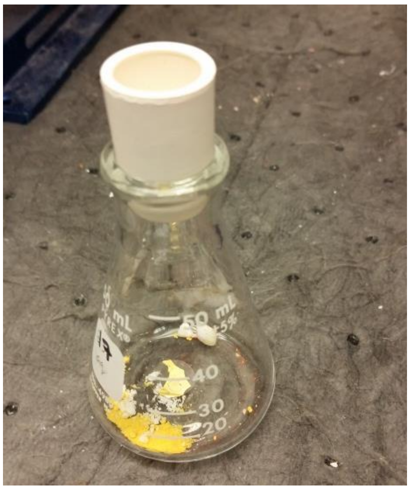

Figure S5: Thin film deposition setup with stirbar, BNAH, and template-stripped gold substrate in preparation. This setup was used for particle lithography experiments.

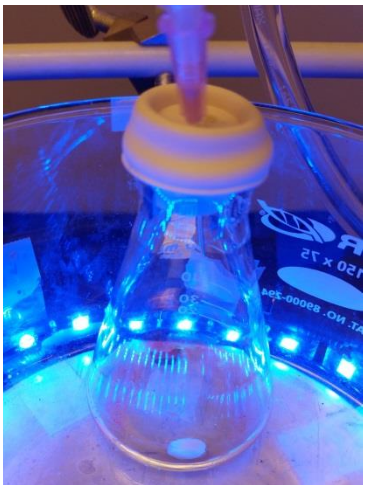

Figure S6: Thin film deposition experiment in progress. This setup was used for particle lithography experiments. Note that the stirbar (at bottom of the image) has been physically separated from the Au substrate (near the LED strip on the other side of the Erlenmeyer flask) to prevent perturbation of the substrate.

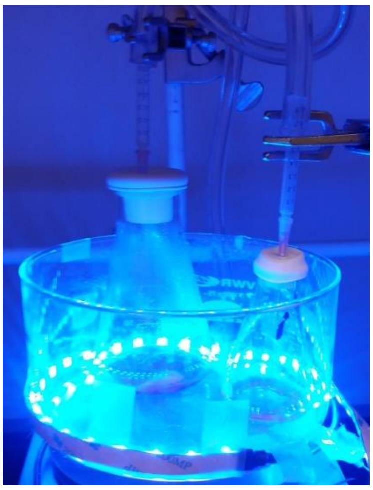

Figure S7: Two thin film deposition experiments in progress. Note the 125 mL flask setup at left that is used for preparation of samples for IRRAS and contact angle goniometry. The 50 mL Erlenmeyer flask at right is used for particle lithography experiments.

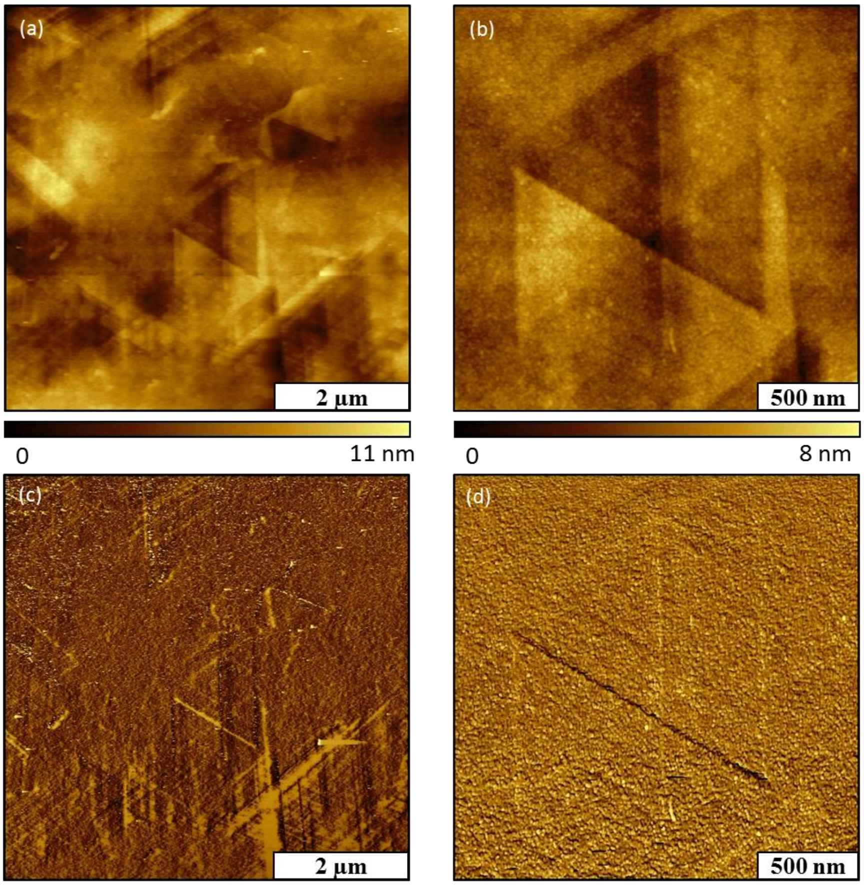

Figure S8: Control sample prepared without adding phthalimide ester. The irradiation of BNAH and $Ru(bpy)_3Cl_2$ in MeCN was conducted for 30 min in the presence of a mask of silicon dioxide mesospheres (500 nm) on Au(111). Tapping mode images were acquired after the removal of the particle mask. (a) Topography image; (b) zoom-in view; (c) corresponding phase image of (a), (d) phase image of (b). No nanostructures were detected, indicating the absence of a thin film. Without phthalimide ester, a thin film is not produced on Au(111). Similarly, multiple attempts at imaging controls performed in the absence of BNAH resulted in no observed nanostructures.

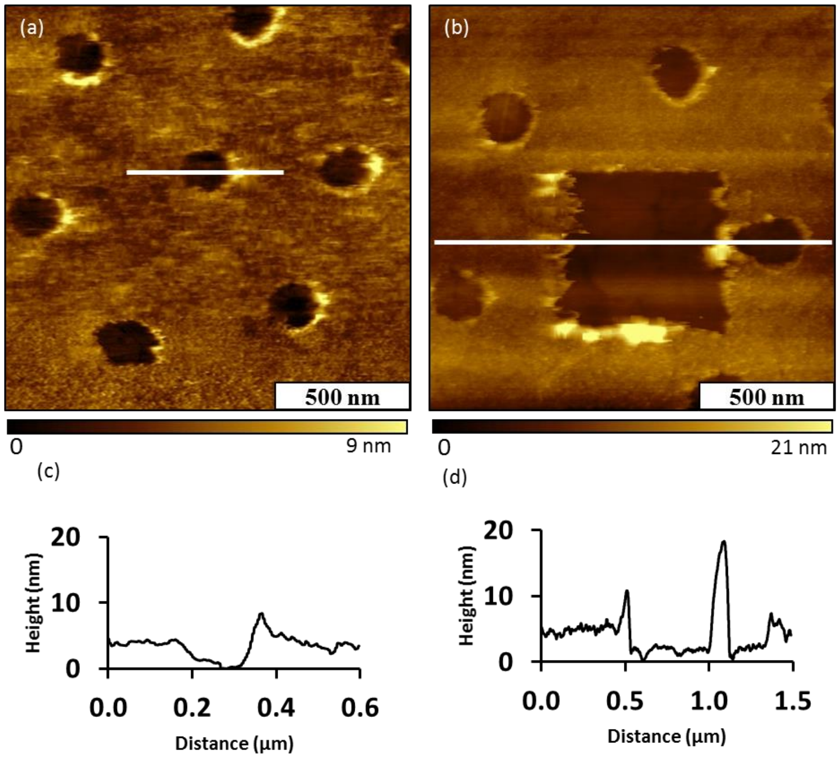

Figure S9: Control sample of Au-Me prepared without applying visible light irradiation. (a) Topograph; (b) nanoshaved area within the Au-Me film; (c) cursor profile for line in (a); (d) cursor profile for line in (b). The sample acquired without visible light irradiation reveals spontaneous deposition on Au(111), with a film measuring 4.0 ± 0.5 nm in thickness. Nanoshaving was conducted using contact mode in an ethanolic solution with 10 nN of applied force. The film prepared without the presence of applied visible light irradiation was easily removed with nanoshaving.

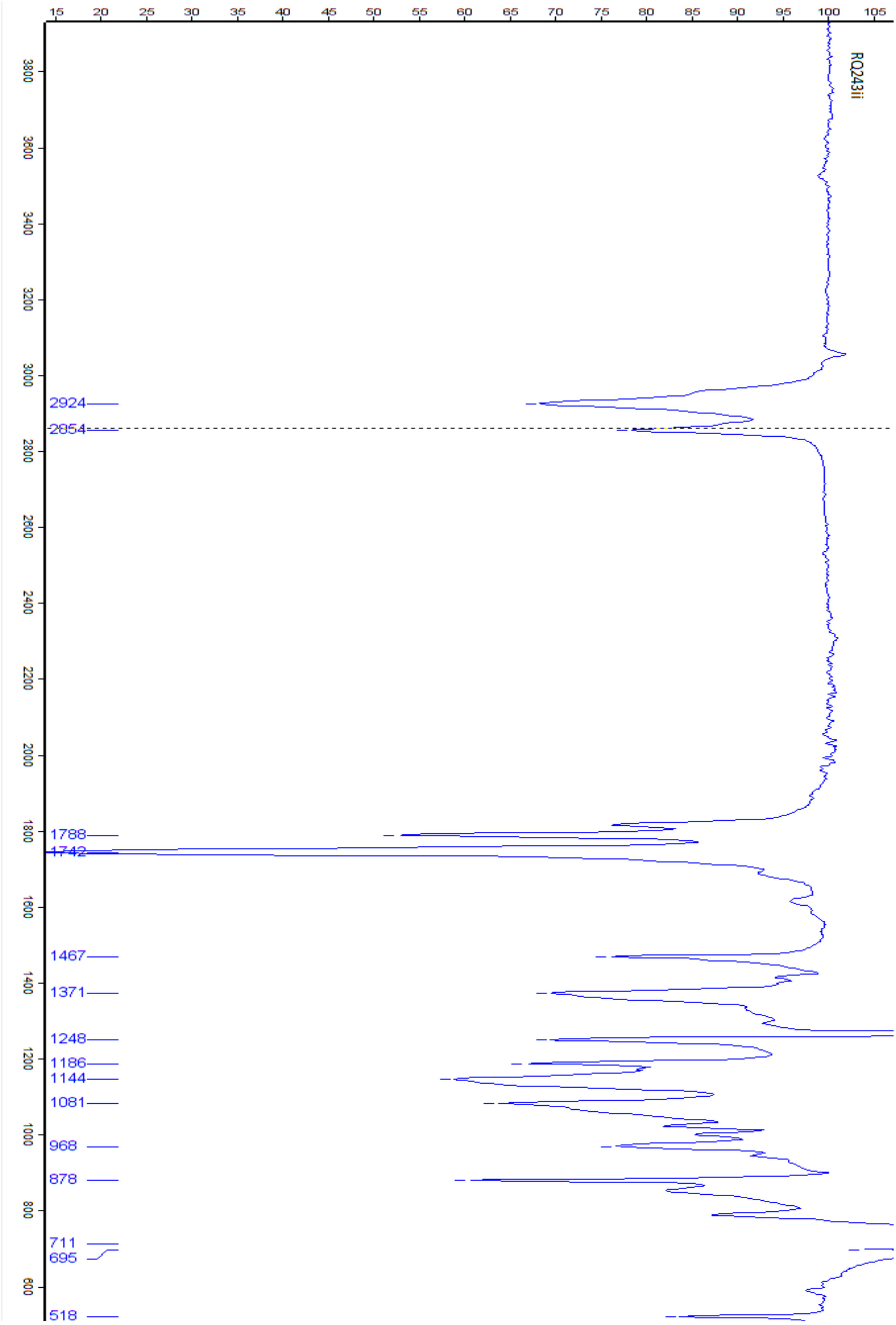

Figure S10: FTIR spectrum of Phth-Me (film).

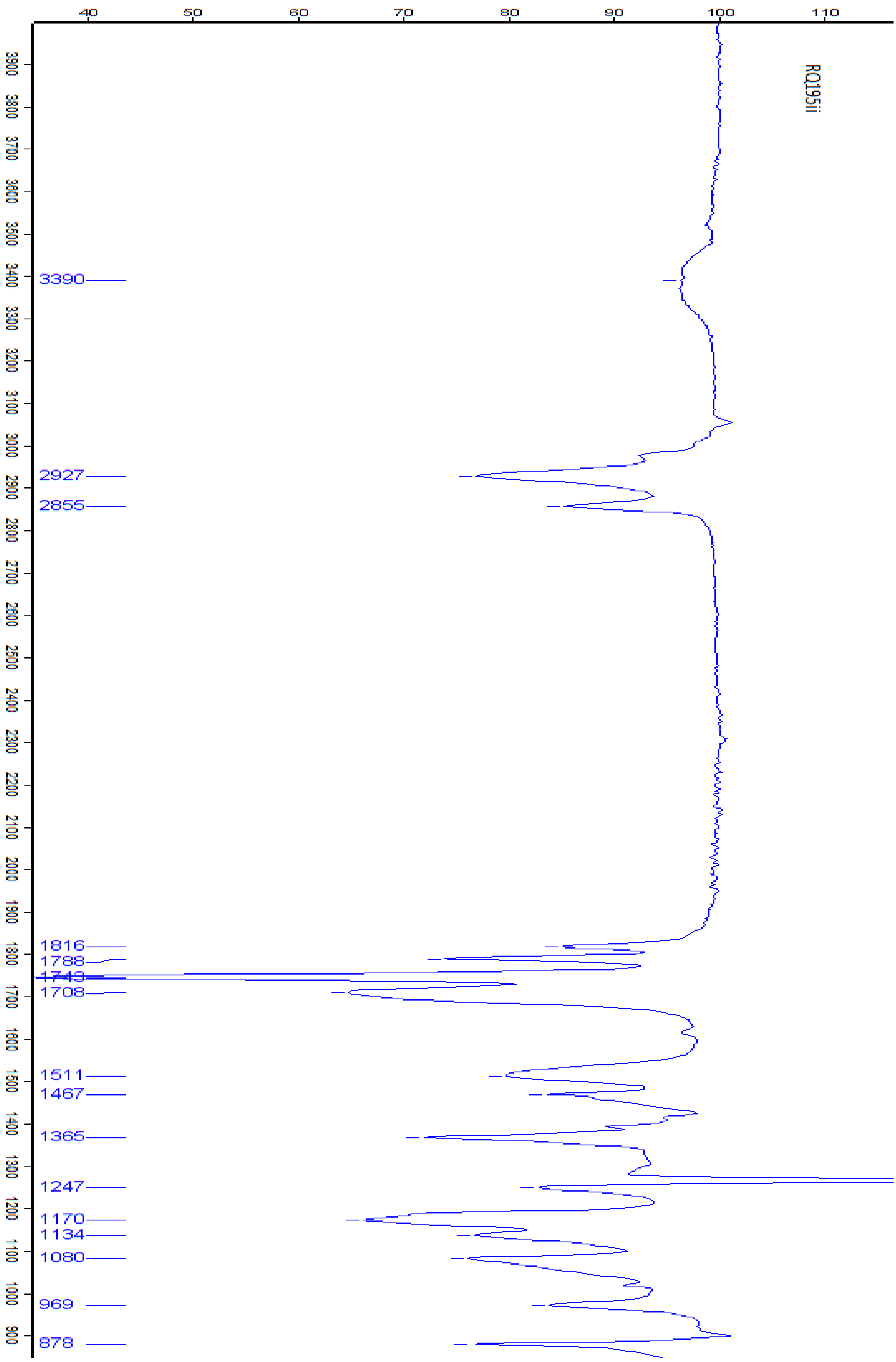

Figure S11: FTIR spectrum of Phth-NHBoc (film).

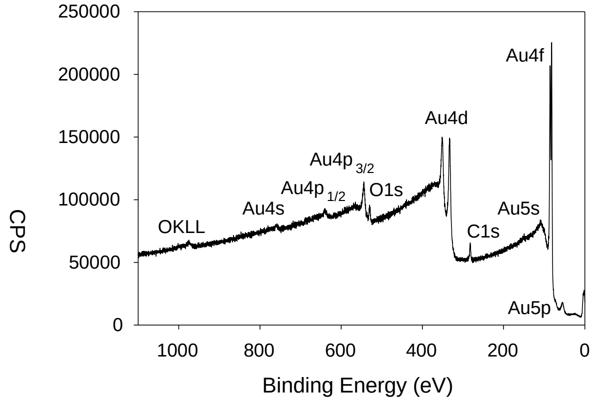

Figure S12: X-ray photoelectron survey spectrum of thin film (Au-Me) under standard conditions.

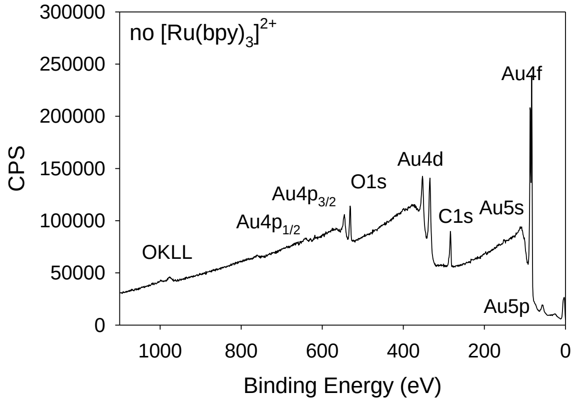

Figure S13: X-ray photoelectron survey spectrum of the deposited Au-Me layer generated without $[Ru(bpy)_3]^{2+}$.

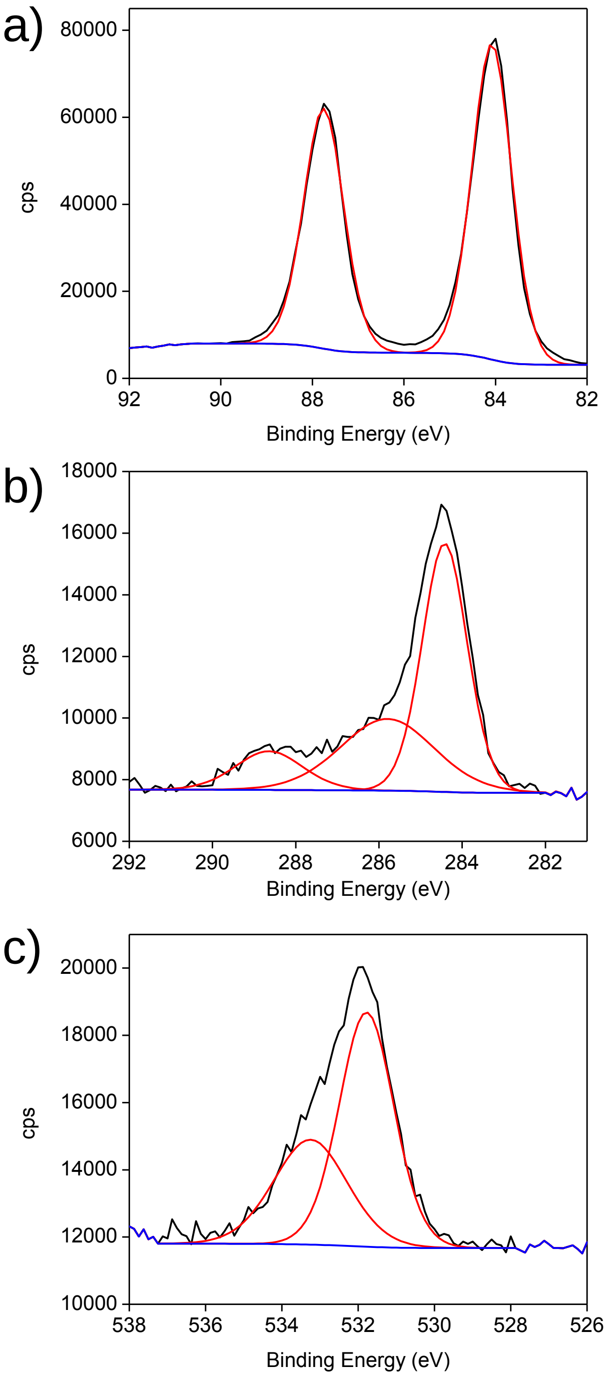

Figure S14: Au 4f, C 1s and O 1s photoelectron spectra of the deposited Au-Me layer prepared without $[Ru(bpy)_3]^{2+}$.

References

1. Schnermann, M. J.; Overman, L. E. *Angew. Chem. Int. Ed.* **2012**, *51*, 9576–9580.

2. Shendage, D. M.; Fröhlich, R.; Haufe, G. *Org. Lett.* **2004**, *6*, 3675-3678.

3. Amara, N.; Mashiach, R.; Amar, D.; Krief, P.; Spieser, S. A. H.; Bottomley, M. J.; Aharoni, A.; Meijler, M. M. *J. Am. Chem. Soc.* **2009**, *131*, 10610–10619.
